# The hepatocyte proteome in organotypic rat liver models and the influence of the local microenvironment

**DOI:** 10.1186/s12953-017-0120-6

**Published:** 2017-06-20

**Authors:** Lucas T. Vu, Sophia M. Orbach, W. Keith Ray, Margaret E. Cassin, Padmavathy Rajagopalan, Richard F. Helm

**Affiliations:** 1 Department of Chemical Engineering, Virginia Tech, Blacksburg, Virginia 24061 USA; 2 Department of Biochemistry, Virginia Tech, Blacksburg, Virginia 24061 USA; 3 School of Biomedical Engineering and Sciences, Virginia Tech, Blacksburg, Virginia 24061 USA; 4 ICTAS Center for Systems Biology and Engineered Tissues, Virginia Tech, Blacksburg, Virginia 24061 USA

**Keywords:** Hepatocyte, Ketogenesis, Liver, Polyelectrolyte multilayer, Proteomics

## Abstract

**Background:**

Liver models that closely mimic the in vivo microenvironment are useful for understanding liver functions, capabilities, and intercellular communication processes. Three-dimensional (3D) liver models assembled using hepatocytes and liver sinusoidal endothelial cells (LSECs) separated by a polyelectrolyte multilayer (PEM) provide a functional system while also permitting isolation of individual cell types for proteomic analyses.

**Methods:**

To better understand the mechanisms and processes that underlie liver model function, hepatocytes were maintained as monolayers and 3D PEM-based formats in the presence or absence of primary LSECs. The resulting hepatocyte proteomes, the proteins in the PEM, and extracellular levels of urea, albumin and glucose after three days of culture were compared.

**Results:**

All systems were ketogenic and found to release glucose. The presence of the PEM led to increases in proteins associated with both mitochondrial and peroxisomal-based β-oxidation. The PEMs also limited production of structural and migratory proteins associated with dedifferentiation. The presence of LSECs increased levels of Phase I and Phase II biotransformation enzymes as well as several proteins associated with the endoplasmic reticulum and extracellular matrix remodeling. The proteomic analysis of the PEMs indicated that there was no significant change after three days of culture. These results are discussed in relation to liver model function.

**Conclusions:**

Heterotypic cell-cell and cell-ECM interactions exert different effects on hepatocyte functions and phenotypes.

**Electronic supplementary material:**

The online version of this article (doi:10.1186/s12953-017-0120-6) contains supplementary material, which is available to authorized users.

## Background

The liver is a highly vascularized organ that performs many essential metabolic functions, including lipid, cholesterol and carbohydrate metabolism as well as the biotransformation of toxins and xenobiotics [1, 2]. The majority of these functions are carried out by the hepatocytes, which constitute about 80% of a mammalian liver’s cellular mass [1, 2]. The non-parenchymal cells (NPCs) of the liver include liver sinusoidal endothelial cells (LSECs), Kupffer cells (KCs), hepatic stellate cells (HSCs), and other cell types such as those involved in the immune response (i.e., natural killer cells) [1, 3]. Hepatocytes are separated from NPCs in vivo by a protein-rich interface that contains extracellular matrix (ECM) proteins, proteoglycans, glycosaminoglycans and other molecules that help maintain the highly differentiated state of hepatic cells [1]. This interfacial region, termed the Space of Disse (SoD), promotes transfer between blood plasma and hepatocytes.

Early efforts to study the liver in vitro mainly used hepatocytes cultured in monolayers (hepatocyte monolayers, HMs) or cultured between layers of type I collagen (collagen sandwich, CS) [4–8]. While HMs begin to lose hepatic phenotypes within four hours [9, 10], CS cultures maintain stable hepatic functions for longer time frames [7, 8, 11]. However, the lack of heterotypic interactions limits the physiological relevance of both HM and CS models. To address this issue, two-dimensional (2D) co-cultures of hepatocytes and NPCs have been developed and were shown to maintain hepatic phenotypes for several weeks [12–18]. Encapsulation of one or more cell types in spheroids has also been shown to enhance hepatic functions such as albumin and urea production and cytochrome p450 (CYP) enzyme activities, and have been established as models for toxicological studies [19–25]. Additionally, primary human hepatocyte (PHH) spheroids cultured for 7 days in chemically-defined media were shown to contain proteomes more similar to that of freshly isolated hepatocytes relative to 2D cultures [26]. This system was also amenable to drug testing and capable of encapsulating NPCs. However, while spheroids and 2D co-cultures facilitate cell-cell interactions as well as permit rapid construct assembly, they do not permit isolation of individual cell types or contain a SoD [27, 28].

An in vitro liver model that incorporated a polyelectrolyte multilayer (PEM) as a SoD mimic was recently described [29–31]. These PEMs were thin films, comprised of alternating layers of chitosan and hyaluronic acid (HA), which exhibit physical and chemical properties similar to the bulk liver and SoD. Models incorporating hepatocytes, LSECs, and KCs were assembled using the PEM to separate the hepatocytes from the NPCs and were arranged to mimic the 3D in vivo architecture. These models maintained hepatic phenotypes and functions throughout a 16-day culture period and exhibited cellular ratios similar to those observed in vivo [30]. Additionally, albumin secretion and CYP1A1 activity were increased in these models as compared to HM, CS and their 2D co-cultures. The system has also recently been used to assess acetaminophen toxicity where strong correlation was obtained between the 3D models and what is observed in vivo [32]. A major advantage to the use of these liver models is the ease of separation of the individual components, permitting selective proteomic analyses. A major disadvantage is the requirement of a cell culturing step to convert isolated hepatocytes into monolayers, a process that permits dedifferentiation, potentially limiting performance. Nonetheless, the assembly and disassembly of 3D models with various concentrations of hepatocytes, PEMs and NPCs, allow insights into cell-cell communication and metabolic processes [29–32].

Previous work with liver models directed towards understanding signaling events have primarily evaluated processes at the transcriptional level [14, 19, 30, 33, 34]. Gene sets related to alcohol, cholesterol, fatty acid, xenobiotic, and carbohydrate metabolism were found to be up-regulated in CS constructs as compared to HMs [33]. The transcriptomes of 3D PEM-based liver models consisting of hepatocytes, KCs and LSECs were also compared against those without KCs [30]. Gene sets related to liver development, proliferation, and the interleukin 4 (IL-4) pathway were up-regulated in hepatocytes when KCs were present. Taken together, these findings support the claim that the local environment modulates both intra- and intercellular processes.

Transcriptional profiling does not monitor changes at post-transcriptional or post-translational levels [35] leading to poor correlations between transcript and protein abundances with time-matched samples [36–40]. While protein abundance levels may not directly reflect enzymatic activities, these measurements provide leads for more targeted approaches such as multiple reaction monitoring (MRM) and enzyme linked immunosorbent assays (ELISA), while also providing information to model and predict cellular biochemistry and physiology through application of the tools of systems biology [41]. Previously published liver proteome studies have evaluated HMs, sandwich cultures of hepatocytes, and immortalized cell lines [11, 36, 39, 42–44]. Protein profiles of freshly isolated primary rat hepatocytes were compared against those sandwiched between either type I collagen or the commercial matrix, Matrigel™, where it was shown that both of these constructs limited dedifferentiation [43], a process that involves the enrichment of structural proteins such as annexin A2, an established biomarker of the process [39, 42]. While these studies provide an initial view of the liver proteome landscape, evaluation of the effects of primary NPCs and the ECM on hepatocyte proteomes have not been explored. We report here on the effects of a collagen/HA PEM and LSECs on hepatocyte proteomes in rat liver models.

## Results

### Liver models overview

**Fig. 1:**
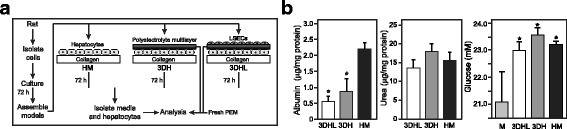
Liver model fabrication and characterization. **a** Experimental overview, (**b**) extracellular albumin (* *p* < 0.05, HM vs. 3DHL and 3DH), urea and glucose (* *p* < 0.05, unconditioned media (M) vs. conditioned media from 3DHL, 3DH and HM) levels for each culture condition after 72 h in culture

A schematic of the assembly and processing workflow is shown in Fig. 1a. Primary rat hepatocytes and LSECs were isolated and cultured as monolayers for 72 hours, at which time three different models were assembled. The HM system was a continuation of the initial culture conditions. The two 3D liver models were assembled by placing PEMs above the hepatocytes as described previously [30]. The 3DH model contained the hepatocytes overlaid with the PEMs only, whereas the 3DHL construct had LSECs seeded above the PEMs. Media was exchanged daily in all models, with constructs cultured for 72 hours. While previous studies have shown that longer culture periods promote increases in hepatic functions, such as albumin and urea synthesis as well as CYP activity [30], we chose to evaluate an early time point in order to establish baseline proteomic measurements in the liver models. Hepatocytes and PEMs were subsequently separated and subjected to processing to peptides that were amenable to bottom-up proteomic analyses.

Albumin secretion, urea production and glucose metabolism were measured to assess liver model function at the end of the culture period (Fig. 1b). Albumin secretion was approximately 4-fold and 2.5-fold higher in the HMs as compared to the 3DHL and 3DH liver models, respectively. This trend is consistent with our previous work and is attributed to hepatocyte stabilization, which takes approximately 2–3 days after addition of the PEMs [29–31]. Urea production and glucose levels were comparable across cultures conditions. Glucose concentrations from spent media of all cultures were significantly higher (*p* < 0.05) than basal levels (22.3 mM). Spent media from the 3DHL, 3DH, and HM models showed increases in glucose of 11%, 13%, and 12% respectively, suggesting that hepatocytes in all cultures were in a gluconeogenic state.

**Fig. 2:**
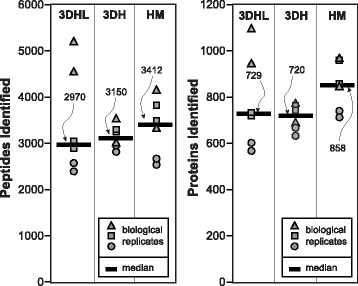
Proteomic overview. Peptides and proteins identified from hepatocyte lysates for each construct. Biological and technical replicates are indicated as well as the median number for each culture condition

The untargeted, bottom-up proteomic analyses utilized data-independent acquisition (DIA). Peptide and protein identifications (Fig. 2) from each construct were performed with PLGS and ISOQuant processing [45] where between 570–1100 proteins were identified among all of the liver cultures at a false discovery rate (FDR) of less than 1% and a requirement of two peptides per protein.

### PEM-containing constructs and HMs have different proteomic signatures

**Fig. 3:**
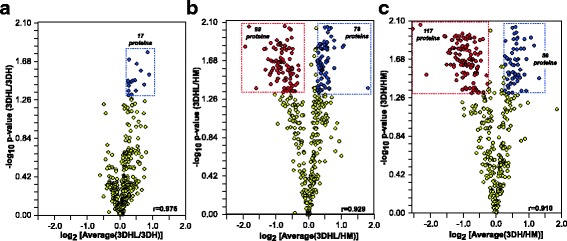
Pairwise proteome comparisons of the three constructs. **a** 3DHL vs. 3DH (**b**) 3DHL vs. HM (**c**) 3DH vs. HM. Proteins that meet established criteria for significance (*p* < 0.05) and abundance ratios (>20%) are shown in blue and red (higher abundance for each pairwise comparison are in blue and those in lower abundance are in red). Pearson correlations for each comparison are indicated in the lower right of each plot

The proteins identified were analyzed using ISOQuant for evaluation of protein abundance ratios using a FDR of less than 1% and at least three peptides per protein. This resulted in the comparison of 447 proteins. Relative abundance ratios were calculated for each pairwise comparison using the ion intensities of the 3 most intense peptides for each protein [45] and are shown in the form of volcano plots (Fig. 3a–c). Proteins that exhibited significant (*p* < 0.05) fold changes of at least 20% are highlighted, a cutoff chosen based on previous experiments performed with in vitro hepatocyte cultures [43, 46]. The protein lists are provided in the Additional file 1: Figure S1 A–C. There were 17 proteins that met the criteria in the 3DHL vs. 3DH comparison (Fig. 3a), all of which were found at higher levels in 3DHL liver models. Interestingly, several of these proteins are associated with the endoplasmic reticulum (ER), ECM remodeling and fibrosis [47–49]. Upon comparing the 3DHL vs. HM comparison (Fig. 3b), 177 proteins were found to be significant with 99 and 78 proteins exhibiting a higher abundance in HM and 3DHL respectively. Additionally, in the 3DH vs. HM comparison (Fig. 3c), 176 proteins were identified as significant with 117 and 59 proteins exhibiting a higher abundance in HM and 3DH respectively. Based on the distributions and the resultant Pearson’s correlation coefficients (Additional file 1: Table S1), the 3DHL and 3DH samples are the most similar to each other.

### PEM-containing constructs favor β-oxidation and ketogenesis

**Fig. 4:**
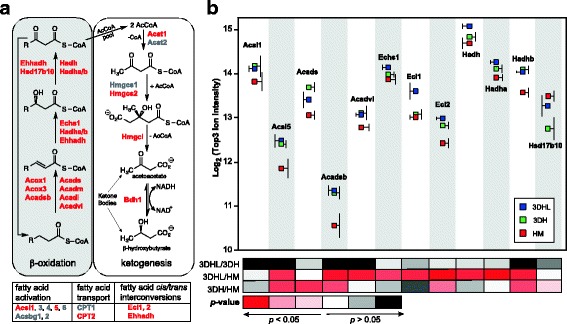
Fatty acid metabolism and ketogenesis are modulated by the PEM and LSECs. **a** Schematic of the main steps in fatty acid β-oxidation. Proteins identified in this study are shown in bold red text. **b** Protein abundances across culture conditions based on the Top3 ion intensities. Ion intensity standard deviations are to the side of each protein/construct pair for clarity purposes and *p*-values for pairwise comparisons are scaled by color

**Fig. 5:**
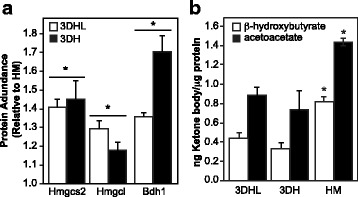
Ketone bodies are produced by all three constructs. **a** Protein abundance ratios for proteins associated with ketogenesis based on the Top3 ion intensities (* *p* < 0.05 HM vs. 3DHL and 3DH). **b** Levels of the two main ketone bodies, acetoacetate and β-hydroxybutyrate, as measured by LC-MS/MS (* p < 0.05 HM vs. 3DHL and 3DH)

The proteins that were significantly different in relative levels in each pairwise comparison were next evaluated for statistically over-represented biological processes using DAVID [50] (Additional file 1: Table S2). Proteins associated with mitochondrial fatty acid β-oxidation and ketone body formation were enriched in the PEM-containing liver models (Figs. 4 and 5). For example, acyl-CoA ligase 5 (Acsl5), which is required for fatty acid activation, was present in higher abundance in 3D liver models over the HMs. The short chain acyl-CoA dehydrogenase enzymes, Acads and Acadsb, also exhibited increases in the 3D models over HMs. While the very long chain (Acadvl) and enoyl-CoA hydratase (Echs1) proteins were more abundant in the 3DHL models relative to HMs, there was no significant difference when 3DH models were compared to HMs. Both enoyl-CoA isomerases (Eci1 and Eci2) exhibited greater abundances in 3DHL liver models as compared to HMs, while only Eci2 also showed a significant increase in 3DH. Hydroxyacyl-CoA dehydrogenase (Hadh) showed significant increases in the 3DHL liver model as compared to HMs while both subunits of the trifunctional enzyme (Hadha and Hadhb) exhibited significant increases in both the 3DHL and 3DH models as compared to HMs. Further interrogation of the lipid metabolism proteins also indicated increases in acyl-CoA oxidase (Acox3) in 3DHL liver models, as well as the peroxisomal bifunctional enzyme (Ehhadh) and peroxisomal 3-ketoacyl-CoA thiolase (Acaa1a) in the 3DHL and 3DH liver models as compared to HMs (Additional file 1: Figure S2). The 3-hydroxyacyl-CoA dehydrogenase type-2 enzyme (Hsd17b10) was also identified but did not exhibit any significant change among any of the pairwise comparisons. These results indicate that both mitochondrial and peroxisomal processes are contributing to β-oxidation in the 3D models.

The formation of ketone bodies (ketogenesis) is reflected by significant changes in three of the four proteins that make up this pathway. Hydroxymethylglutaryl-CoA synthase (Hmgcs2), hydroxymethylglutaryl-CoA lyase (Hmgcl) and β-hydroxybutyrate dehydrogenase (Bdh1) all show increases in 3D models as compared to HMs (Figs. 4a and 5a). The changes in proteins associated with ketogenesis prompted evaluation of ketone body concentrations in the spent cell culture media using a targeted LC-MS/MS approach [51]. Contrary to protein abundances where the PEM-containing liver models exhibited higher levels of these proteins, concentrations of acetoacetate and β-hydroxybutyrate in the cell culture media were significantly higher in the HMs as compared to the 3D models (Fig. 5b).

### HMs contain higher levels of several glycolytic/gluconeogenic enzymes

**Fig. 6:**
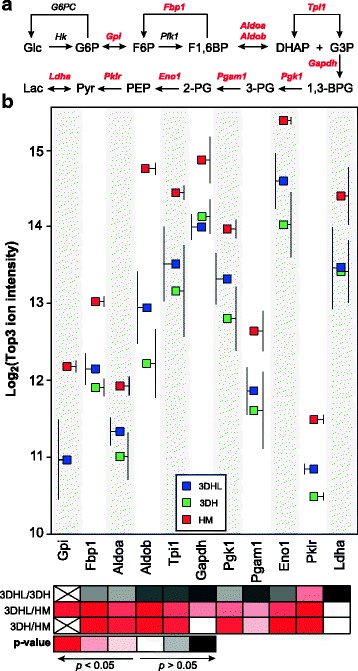
Proteins associated with glucose processing are more abundant in HMs. **a** Pathway with proteins identified in this study shown in red. **b** Protein abundances based on the Top3 ion intensities. Ion intensity standard deviations are to the side of each protein/construct pair for clarity purposes and *p*-values for pairwise comparisons are scaled by color. Gpi was not detected in all 3DH analyses and thus not included

Glucose concentrations in spent culture media were higher than basal levels (Fig. 1b), indicative of a gluconeogenic state. Eleven enzymes involved in glycolysis/gluconeogenesis exhibited significantly increased levels in the HM cultures (Fig. 6). These include glucose 6-phosphate isomerase (Gpi), fructose-1,6-bisphosphatase (Fbp1, a key enzyme for gluconeogenesis), fructose bisphosphate aldolase A and B (Aldoa and Aldob), triosephosphate isomerase (Tpi1), glyceraldehyde 3-phosphate dehydrogenase (Gapdh), phosphoglycerate kinase 1 (Pgk1), phosphoglycerate mutase 1 (Pgam1), alpha-enolase (Eno1) and pyruvate kinase isozymes R/L (Pklr). No difference was observed in lactate dehydrogenase A (Ldha) abundance among the culture conditions. However, 3DHL liver models showed significantly higher levels of Pklr as compared to the 3DH models. Based upon the increased level of Fbp1, extracellular glucose levels in HMs would be predicted to be higher than in the PEM-containing models. However, glucose production rates are under control of both allosteric effectors as well as thermodynamic constraints [52]. Thus, as with the ketone body measurements, the extracellular glucose concentrations do not directly correlate with the predictions based on protein abundance. Nonetheless, all liver constructs provide local environments where the hepatocytes release glucose even though the extracellular media concentration is significantly higher than what is observed in vivo [53].

### Structural and trafficking proteins in HMs

**Fig. 7:**
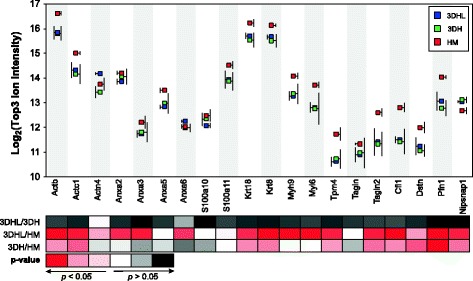
PEMs and LSECs mitigate production of proteins associated with hepatocyte dedifferentiation. Structural and migratory protein abundances across culture conditions based on the Top3 ion intensities. Ion intensity standard deviations are to the side of each protein/construct pair for clarity purposes and *p*-values for pairwise comparisons are scaled by color

A hallmark of hepatocyte dedifferentiation is the modification of cell shape and structure [7, 8, 29]. Several structural and trafficking proteins were found in higher abundance in HMs relative to the 3D liver models (Fig. 7). Actin (Actb and Actc1), S100-A11, keratins (Krt 8 and Krt18), tropomyosin (Tpm4), transgelin (Tagln2), cofilin (Cfl1), destrin (Dstn) and profilin (Pfn1) all exhibited increased levels in HM relative to both liver models containing PEMs. Annexins (Anxa 2, 5, and 6) and myosins (Myl6 and Myh9) all exhibited higher abundance in HMs as compared to 3DHL models. Only 4-Nitrophenyl phosphatase domain and nonneuronal SNAP25-like protein homolog1 (Nipsnap1) showed increased levels in both 3DH and 3DHL models as compared to HMs. Alpha actinin 4 (Actn4) showed increased levels in 3DHL as compared to 3DH. As a whole, the increased levels of structural proteins observed in the HM cultures are indicative of changes in hepatocyte morphology and migration, possibly indicating of dedifferentiation. Future studies evaluating the temporal response of these proteins will need to be conducted in order to validate this claim.

### 3DHL models exhibit higher levels of proteins associated with drug metabolism

**Fig. 8:**
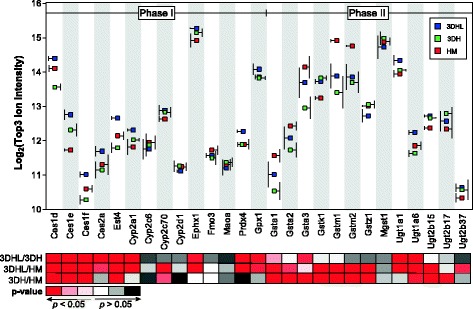
Comparison of drug metabolism related proteins among all constructs. Drug metabolism related protein abundances across culture conditions based on the Top3 ion intensities. Proteins are organized based upon class: Phase I and II enzymes. Ion intensity standard deviations are to the side of each protein/construct pair for clarity purposes and *p*-values for pairwise comparisons are scaled by color

KEGG pathways related to drug metabolism were enriched in the 3DHL liver models (Additional file 1: Table S2 and Fig. 8). Overall, 27 Phase I and Phase II biotransformation enzymes were quantified using ISOQuant. The Phase I enzymes include carboxylesterases (Ces and Est), Cyps, epoxide hydrolase (Ephx), dimethylaniline monooxygenase (Fmo), amine oxidase (Maoa), peroxiredoxin (Prdx4), and peroxidase (Gpx1). Phase II enzymes include glutathione-S-transferase (Gst) and UDP-glucuronosyltransferase (Ugt) isozymes. Out of the 27 Phase I and Phase II biotransformation proteins quantified, 16 exhibited significantly increased levels in 3DHL models as compared to HMs indicating that both the LSECs and PEMs have an effect. In order to evaluate the effects of just the PEMs on biotransformation enzyme levels, 3DH liver models were compared to HMs. Only 6 of the 27 enzymes exhibited increased abundance in 3DH as compared to HMs indicating that the combination of LSECs and PEMs play a more dominant role in modulating protein abundance. This was verified by comparing 3DHL to 3DH models where 14 proteins exhibited higher levels in 3DHL as compared to 3DH. These results support the claim that biotransformation enzyme abundances within the liver models are enhanced by the presence of LSECs.

### Proteomic analysis of the PEMs shows limited matrix remodeling

The ECM plays a crucial role in maintaining hepatic function [19]. While previous studies demonstrate that PEM containing liver models maintain hepatic functions and phenotypes for extended time periods relative to HMs [29–31], in-depth studies on the PEM proteome itself have not been performed on liver models. PEMs were isolated from the 3DHL and 3DH liver models at the same time point as the hepatocytes. These materials along with unconditioned PEMs (“fresh” PEMs) and analyzed by LC-MS/MS after processing to peptides. Protein identifications were performed using a database that contained proteins associated with the ECM obtained from the UniProt database. To assess for possible contamination due to the incomplete removal of LSECs, a separate analysis of the PEMs was performed using the rat proteome (data not shown). Only collagen components were identified suggesting sufficient removal of the LSECs from the PEMs. The proteins that were identified from the PEM analyses were then quantified using peptides unique to each identified protein, are these results are shown in Table 1. The PEMs are composed predominantly of Type I collagens (alpha-1 and alpha-2 forms), with those from the 3DHL and 3DH cultures exhibiting no significant proteomic differences as compared to the fresh PEM proteome. These results indicate that substantial changes in the proteome of the PEMs are limited after 3 days of culture.

**Table 1:** Proteins identified from conditioned (3DHL, 3DH) and unconditioned (FP) PEMs

Description	UniProt ID	Unique peptides	3DHL/FP (*p*-value)	3DH/FP (*p*-value)
Collagen alpha-1 (I), Col1a1	P02454	258	1.34 ± 0.18 (0.64)	1.30 ± 0.06 (0.46)
Collagen alpha-2 (I), Col1a2	P02466	198	1.06 ± 0.08 (0.72)	1.08 ± 0.05 (0.59)
Collagen alpha-1 (III), Col3a1	P13941	173	1.07 ± 0.06 (0.78)	1.17 ± 0.13 (0.56)
Protein Col28a1	D3ZN64	126	1.36 ± 0.08 (0.63)	1.56 ± 0.21 (0.76)
Protein Col4a2	F1M6Q3	125	1.43 ± 0.07 (0.58)	1.35 ± 0.08 (0.49)
Collagen alpha-1 (II), Col2a1	P05539	74	1.21 ± 0.02 (0.82)	1.21 ± 0.14 (0.94)
Collagen alpha-1 (XI)	P20909	69	1.03 ± 0.18 (0.92)	1.19 ± 0.49 (0.70)
Procollagen alpha-2 (XI), Col11a2	Q6MGB2	61	1.50 ± 0.16 (0.67)	1.58 ± 0.16 (0.44)
Procollagen alpha-1 (VII), Col7a1	D3ZE04	54	0.71 ± 0.33 (0.63)	0.80 ± 0.45 (0.69)
Protein Col4a5	F1LUN5	50	1.26 ± 0.02 (0.76)	1.24 ± 0.03 (0.62)
Protein Col5a2	F1LQ00	44	0.66 ± 0.58 (0.66)	0.40 ± 0.52 (1.00)
Protein Col4a1	F1MA59	39	0.74 ± 0.18 (0.65)	0.73 ± 0.23 (0.57)
Collagen alpha-1 (XXVII), Col27a1	Q80ZF0	31	0.95 ± 0.17 (0.78)	0.94 ± 0.02 (0.57)
Protein Col10a1	A0A0G2K7A5	29	1.16 ± 0.05 (0.70)	1.21 ± 0.01 (0.54)
Procollagen alpha-1 (V), Col5a1	G3V763	15	1.47 ± 0.11 (0.63)	1.53 ± 0.10 (0.44)
Protein Col14a1	D3ZZT9	14	0.51 ± 0.09 (0.70)	0.44 ± 0.06 (0.52)
Fibromodulin	P50609	5	0.32 ± 0.06 (0.95)	0.29 ± 0.02 (0.83)

Ratios were calculated based on the Top 3 method as described in the experimental section. Unique peptides represent the sum total of identifications across all samples

## Discussion

This study was designed to measure the effect of the local environment on the primary rat hepatocyte proteome of three different culture conditions. Distinct proteomic profiles were observed with hepatocytes from both the 3D models relative to HMs (Figs. 2 and 3), with the most dramatic differences related to fatty acid β-oxidation and ketogenesis (Figs. 4 and 5), glycolysis/gluconeogenesis (Fig. 6), and drug metabolism (Fig. 8). It was also determined that structural and trafficking proteins as well as those associated with cellular migration were increased in the HMs relative to the 3D liver models (Fig. 7).

The majority of proteins in the fatty acid β-oxidation (mitochondrial and peroxisomal) and ketogenesis pathways were found in higher abundances in the 3DHL and 3DH liver models when compared to HMs (Figs. 4 and 5). When intracellular concentrations of glucose in the liver are low, increases in fatty acid β-oxidation occur resulting in production of acetyl-CoA for feeding the Krebs/TCA cycle and subsequently oxidative phosphorylation. Prolonged low intracellular glucose levels and depleted glycogen stores in hepatocytes leads to ketogenesis for transport of ketone bodies to extrahepatic tissues, where conversion back to acetyl-CoA occurs for utilization as an energy source. Increases in ketogenic proteins prompted the quantification of ketone bodies in the cell culture media. These analyses confirmed the presence of ketone bodies in spent cell culture media for all constructs. However, in contrast to the protein abundance data, HMs exhibited higher levels of both acetoacetate and β-hydroxybutyrate relative to the 3DHL and 3DH liver models (Fig. 5). This discrepancy can be attributed to the fact that protein abundances may not account for changes in enzyme activity due to protein-protein interactions and/or post-translational modifications. Bdh1, for example, requires lipid binding for full enzymatic activity [54], and is known to be *O*-GlcNAcylated [55], a post-translational modification directly associated with cellular energy status [56]. Additionally, it is also possible that the PEM limits diffusion of these molecules. Further studies will be needed to evaluate these hypotheses. Nonetheless, the models are producing ketone bodies with the proteomic data indicating fatty acid β-oxidation as the energy source.

All three liver models were synthesizing glucose (Fig. 1b) despite the fact that the extracellular level was more than twice that of physiological concentrations [53]. Ketone body production combined with gluconeogenesis are features of a diabetic state [57]. A recent study using a micro-patterned co-culture of human hepatocytes and murine fibroblasts in the presence of insulin [58] demonstrated increased lipid accumulation in hyperglycemic cultures (25 mM glucose) after 10–18 days along with erratic glucose consumption relative to normo- and hypo-glycemic conditions in the presence of insulin. Evaluation of the effects of insulin levels on these systems also indicated that hyperglycemic conditions led to insulin resistance over time. Others studies have shown that HM and CS cultures lose their ability to perform glycolysis and gluconeogenesis after 3–4 days in culture even when stimulated with insulin and glucagon [59, 60].

While glucose levels increased in spent culture media from all constructs, only the HM proteome exhibited higher levels of proteins related to glycolysis and gluconeogenesis (Fig. 6). These contrasting data are most likely due to glucose metabolism being allosterically and hormonally regulated. As a case in point, Fbp1 catalyzes the rate-limiting step in gluconeogenesis and is synergistically inhibited by both fructose 2,6-bisphosphate and adenosine monophosphate (AMP) [52, 61]. With regards to hormonal control, the hepatocyte medium used in this study contained FBS, glucagon and insulin. Given that glucagon is typically produced in vivo as a response to low glucose levels, the supplement may be overriding the effects of insulin, placing the cells in a state perceived as starvation. Glucagon has been shown to increase hepatic glucose output, mostly via glycogenolysis, and inhibits glucose uptake [62]. Further studies will be required to determine the roles of gluconeogenesis and glycogenolysis in controlling intracellular and extracellular glucose levels in liver models. Future studies should routinely include glucose measurements to assess the temporal status of glucose metabolism. The composition of the cell culture media should also be systematically evaluated as changes in hormone levels (a decrease in glucagon concentrations for example) may provide more physiologically relevant metabolic performance.

It has been shown that transcriptional changes in HMs occur as early as 4 hours, essentially marking the beginning of dedifferentiation [9, 10]. In addition to changes in the transcriptome, after seven days in culture alterations in hepatocyte morphology are observed, with corresponding increases in actin stress fibers [7, 8, 29]. Several studies have characterized the proteomic changes associated with this process [11, 36, 39, 42, 43]. Azimifar and coworkers evaluated murine HMs at time points similar to those used in our work [39]. The proteomic changes reported as being related to dedifferentiation were increases in structural and trafficking proteins, such as those in higher abundance in our HM construct (Fig. 7). Annexins are multi-functional proteins often associated with cancer growth and membrane trafficking [63]. In our study, three annexins (Anxa2, Anxa5 and Anxa6) exhibited greater abundance in HM relative to the 3DHL liver models, suggestive of dedifferentiation. Additionally, S100-A11, which facilitates cell-cell contact through annexin [64], was also identified as being significantly increased in HMs as compared to 3DHL models. Other structural proteins identified in common in both studies were keratins (Krt 8), actins (Actb and Actc1) and alpha actinin 4 (Actn4). While Krt18 was not identified in the study performed by Azimifar and coworkers, keratins are cytoskeletal intermediate filament proteins that have been observed to increase in levels in dedifferentiated hepatocytes [65]. Proteins related to cell migration and motility were also increased in HMs, including myosins (Myl6 and Myh9), tropomyosin (Tpm4), transgelin (Tagln2), cofilin (Cfl1), destrin (Dstn) and profilin (Pfn1). Myosins, tropomyosins and transgelins regulate the contraction and movement of cells [65]. Silencing of transgelin proteins has been shown to inhibit the migration and invasion of huh7 (hepatocarcinoma) cells [66]. Cofilin, destrin and profilin are proteins that regulate actin dynamics by initiating and inhibiting the polymerization, depolymerization and recycling processes [67]. As previous observations have shown that HMs exhibit flattened and larger morphologies indicative of cell spreading and increased contractility [7], these results provide a foundation for understanding how structural and cell migration proteins contribute to the dedifferentiation in hepatocytes. However, a temporal evaluation is needed to provide further insight.

While most of the structural and migration proteins exhibited increases in HMs, Actn4 and Nipsnap1 exhibited increases in the 3D liver models. Alpha actinin proteins such as Actn4 are involved in the cross linking of actin stress fibers [68], which have been shown to form during dedifferentiation. However, previous studies have also shown an increased and sustained mRNA expression of alpha actinin between 24 and 48 hours after partial hepatectomy [69]. An increased abundance of Actn4 in the 3DHL liver models relative to 3DH models and HMs indicates that the 3DHL liver models exhibit similarities to what has been observed during liver regeneration. Nipsnap1 exhibited increased abundance in both liver models relative to HMs. While NipSnap1 is usually referred to as a trafficking protein, a murine liver knockout showed increased production of amino acids, nucleotides, ketone bodies and β-oxidation, with decreased fatty acid production [70]. These findings suggest a regulatory role of Nipsnap1 in core metabolic flux.

As an initial survey into the ECM proteome, PEMs from the 3DHL and 3DH liver models were analyzed along with an unused PEM (FP) to assess changes upon cell culture and potential ECM biosynthesis. The results showed no significant changes in composition of the PEMs from 3DHL and 3DH liver models as compared to FPs (Table 1). We conclude from these results that after 72 hours only minor changes in the ECM occurred, and these changes were too small to detect in combination with the PEM collagen background. We hypothesize that longer culture periods will allow more time for the cells to remodel the local PEM environment, making the region more conducive to maintaining liver function.

One main objective of this effort was to assess any potential impacts of LSECs on the hepatocyte proteome. There were 17 proteins significantly higher in abundance in the 3DHL hepatocytes as compared to the 3DH cells (Fig. 3a). Interestingly, 7 of these proteins are associated with the ER and ECM remodeling: calnexin (Cnx), peptidyl-prolyl cis-trans isomerase B (PPIase B), endoplasmic reticulum resident protein 29 (Erp29), protein disulfide-isomerase A4 (Pdia4), protein disulfide-isomerase (Pdi), 78 kDa glucose-regulated protein (Grp78) and UDP-glucuronosyltransferase 1–6 (Ugt1a6). High levels of proteins such as Cnx, Erp29, and Grp78 are associated with ER stress [71–73]. In addition, the glucose-regulated proteins Grp78 and Grp94 and Pdi are all involved in the post-translational modifications of type I collagen [48]. Cyclophilin J, which is a member of the PPIase family, was identified as a protein that promotes the cell cycle transition from the G1/S phase in human hepatocarcinoma [74]. Inhibition of cyclophilin has also been shown to have an anti-fibrotic effect suggesting a regulatory role in ECM remodeling [47, 49]. Taken together, the changes in the ER proteome observed in the 3DHL models are potentially associated with the initial phase of ECM remodeling, and may require the presence of LSECs. Additional experiments utilizing longer culture periods will permit full assessment of this observation.

The biotransformation of drugs, xenobiotics and normal cellular waste products is a critical liver function. The 3DHL liver models exhibited significantly higher levels of carboxylesterases and other Phase I and Phase II detoxification enzymes as compared to both 3DH and HM models (Fig. 8). Carboxylesterases are a class of Phase I and lipid processing enzymes that hydrolyze molecules containing esters, amides, thioesters and carbamates [75]. Increased extracellular glucose concentrations (25 mM) in cultured primary mouse hepatocytes leads to increased levels of Ces1d (76% identical to Ces1d in rat), with the increase linked to the farnesoid X receptor (FXR). This transcriptional regulator is typically activated by lipophilic compounds such as bile acids, providing global control of lipid and glucose metabolism [76, 77], where high glucose concentrations favor lipogenesis and glycogen formation. *Ces1g* knockout mice are obese and exhibit non-alcoholic fatty liver disease, a phenotype that can be rescued solely by the addition of polyunsaturated fatty acids [78]. These studies highlight the role of carboxylesterases in regulating core lipid metabolism. As the carboxylesterases were present in significantly higher amounts in the 3DHL models relative to the 3DH and HM models, it is possible that these enzymes were produced due to the presence of LSECs in order to modulate fatty acid and glucose metabolism.

When hepatocytes are cultured as monolayers, the local environment is not conducive to cell stability as a region of every cell will face bulk media. Both the basal and apical polarities are eventually lost due to the lack of proper cell-ECM and cell-cell interactions [79]. As a result, the synthesis of structural “stabilizing” components, such as annexins are elicited. One drawback of the models described in this work is the requirement of a confluent hepatocyte monolayer prior to assembly, a process that requires growth as monolayers (Fig. 1a). As hepatocyte dedifferentiation is initiated within hours of isolation, this culturing step may be limiting construct performance potential. Efforts at reducing or eliminating this step may dramatically enhance performance. Nonetheless, the presence of an appropriate matrix, such as found in a PEM, CS cultures, or spheroids, hepatocyte polarity is maintained, mitigating structural changes. A non-supportive environment will eventually lead to loss of phenotype, either through structural changes, the inability to fully utilize the media components, and/or the production of toxic compounds that cannot be effectively eliminated.

Taken together, our results show that the 3D liver models and HMs exhibit shifts towards fatty acid and glucose metabolism, respectively. Additionally, the presence of LSECs led to increases in detoxification enzyme abundance, indicating that LSECs and PEMs have an effect on liver metabolism. Finally, increases in structural and migratory proteins, a sign of hepatocyte dedifferentiation, was observed in HMs, highlighting the need for cell-cell and cell-ECM interactions for maintenance of a functional state.

## Conclusions

Mass spectrometry-based proteomic analyses were performed to assess the effects of PEMs and LSECs on hepatocyte function in three different liver models. The systems were shown to produce urea, albumin and glucose, with shifts to fatty acid metabolism and ketone body production as determined by both proteomics as well as a quantitative assessment of extracellular ketone body concentrations. Liver models that included PEMs and/or LSECs exhibited higher levels of proteins associated with lipid metabolism, and more specifically fatty acid β-oxidation and ketogenesis. It was also determined that HMs exhibited increased abundance of enzymes related to glucose metabolism. Although minimal changes in the PEM were found, the structure limited the abundance of structural and migratory proteins associated with hepatocyte dedifferentiation. Several carboxylesterases exhibited a dependence on the presence of LSECs providing a glimpse into intercellular signaling processes. Taken together, these results suggest that the heterotypic cell-cell and cell-ECM interactions exert different effects on heptocyte functions and phenotypes, which consequently affect metabolism. Future studies that compare hepatocyte and NPC proteomes to those of freshly isolated cells and spheroids will aid in determining key processes involved in the long-term stability of liver models.

## Methods

### Chemicals and reagents

Cell culture supplies including Dulbecco’s Modified Eagle Medium (DMEM), phosphate-buffered saline (PBS), penicillin, streptomycin, human plasma fibronectin and trypsin (0.25% EDTA) were purchased from Thermo Fisher Scientific (Grand Island, NY). HEPES (4-[2-hydroxyethyl] piperazine-1-ethanesulfonic acid), ethylenediaminetetraacetic acid (EDTA), glucagon, glutaraldehyde, hyaluronic acid (HA), hydrocortisone, Percoll®, Type IV collagenase, chloroform, ammonium bicarbonate (AmBic), urea, trifluoroacetic acid (TFA), sodium 3-hydroxybutyrate, lithium acetoacetate, mass spectrometry grade trypsin and endothelial cell growth supplement were purchased from Sigma Aldrich (St. Louis, MO). Mass spectrometry grade lysyl endopeptidase (Lys-C) was purchased from Wako (Richmond, VA). LC-MS grade solvents were purchased from Spectrum Chemicals (New Brunswick, NJ). All other chemicals, unless noted otherwise, were purchased from Fisher Scientific (Pittsburgh, PA).

### Type I collagen isolation

Type I collagen was isolated from rat tail tendons as previously reported [8, 80]. Briefly, tendons were dissected from tails and dissolved in a 3% (v/v) acetic acid solution overnight at 4 °C. The solution was subsequently passed through cheesecloth filters and centrifuged at 13,000 *x g* for 2 hours at 4 °C. The supernatant was precipitated using 30% (w/v) NaCl and the resulting precipitate was dissolved in 0.6% (v/v) acetic acid for 48 hours at 4 °C. The solution was dialyzed against 1 mM HCl and sterilized by chloroform. The concentration of collagen was determined by measuring the optical density at 280 nm.

### PEM assembly

Type I collagen (cationic) and hyaluronic acid (HA, anionic) were used for PEM assembly described previously [30, 81]. Collagen was diluted in 1% (v/v) acetic acid and HA was dissolved in ultrapure water (resistivity > 18 MΩ-cm). Both solutions were maintained at a concentration of 1.5 mg/mL and a pH of 4.0. PEMs were assembled on a hydrophobic polytetrafluoroethylene (PTFE) substrate (McMaster-Carr, Atlanta, GA) using a robotic deposition system (NanoStrata, Tallahassee, FL). The PTFE substrates were sonicated in toluene for 1 hr, rinsed in ultrapure water, and dried overnight. Water contact angle measurements were used to verify the hydrophobicity of the substrate and ranged between 110°–115° (averaged over 10 measurements per substrate). Deposition times of 30 min were used for each polyelectrolyte with a 10-minute rinse in ultrapure water, maintained at a pH of 4.0, between each deposited layer. The PEMs used in these studies consisted of 15 bilayers, with one bilayer defined as the deposition of one cationic and one anionic layer. The assembled PEMs were then cross-linked with 8% (w/v) glutaraldehyde for 30 sec, rinsed in ultrapure water for 48 hours, dried, and stored at room temperature. Immediately prior to their use, PEMs were sterilized under germicidal UV for 1 hour.

### Isolation and culture of hepatocytes and LSECs

The primary hepatocytes and LSECs were harvested from two adult female Lewis rats that weighed between 170 and 200 grams. Animal care and surgical procedures were conducted as per procedures approved by Virginia Polytechnic Institute and State University’s Institutional Animal Care and Use Committee. Hepatocytes were isolated using a two-step collagenase perfusion method as previously described [29–31, 82]. Viability of isolated hepatocytes were ≥ 97% as determined by trypan blue exclusion. Hepatocytes were cultured on type I collagen coated 12-well plates at a density of 500,000 cells per well. Hepatocyte cultures were maintained in high glucose (25 mM) DMEM supplemented with 10% (v/v) heat inactivated fetal bovine serum (FBS), 200 U/mL penicillin, 200 μg/mL streptomycin, 0.5 U/mL insulin (MP Biomedicals, Solon, OH), 4 nM glucagon, and 16 μM hydrocortisone. LSECs were purified from the same isolation using a differential adhesion method [30, 31] and were cultured on a fibronectin coated flask for 72 hours. LSECs were maintained in endothelial cell medium consisting of Media 199 supplemented with 10% (v/v) heat inactivated FBS, 100 U/mL penicillin, 100 μg/mL streptomycin, 2 mM L-glutamine, and 30 μg/mL endothelial cell growth supplement. Both hepatocytes and LSECs were cultured in their respective media for 72 hours with media exchanges daily.

### Liver model assembly

Liver model assembly occurred 72 hours after isolation to allow hepatocytes to spread and form a confluent layer on the type I collagen gel [29–31]. The HM construct simply involved replacing the media with fresh hepatocyte media (1 mL). The two PEM-based liver models were assembled as described previously [30]. A detachable PEM was overlaid above the hepatocytes, and subsequently hydrated with hepatocyte media for 1 hour. The addition of hepatocyte media to this system provided the 3DH model. The third construct, termed 3DHL, followed the 3DH assembly process, except that after PEM hydration, 25,000 LSECs were seeded above the PEM. This seeding density was chosen based upon previous optimization efforts [30]. All three constructs were then maintained for an additional 72 hours, with media exchanged every 24 hours. Spent culture media were collected and snap frozen in liquid nitrogen and stored at−80 °C.

### Hepatocyte isolation and processing of lysates

Hepatocytes were separated from both the 3D liver models by lifting the PEM from the culture. The hepatocytes on the underlying gel were washed with PBS and released from the collagen gel using a 0.1% (w/v) collagenase solution in Krebs-Ringer buffer. HM cultures were subjected to the same collagenase digestion. Hepatocytes were collected by centrifugation at 800 *x g* for 2 min and were subsequently lysed in a buffer containing 4.3 mM Tris–HCl, 660 μM Tris-Base, 500 μM EDTA, 15 mM NaCl, 350 μM SDS, 0.1% (v/v) Triton X-100, 310 μM sodium azide, 150 μM sodium vanadate, and was supplemented with a protease inhibitor (PI) cocktail (Sigma-Aldrich; 104 mM 4-(2-aminoethyl) benzenesulfonyl fluoride hydrochloride (AEBSF), 80 μM aprotinin, 4 mM bestatin, 1.4 mM epoxide 64 (E-64), 2 mM leupeptin and 1.5 mM pepstatin A). Protein concentrations were determined using a commercial BCA protein assay kit (Thermo Fisher Scientific). LC-MS grade methanol was added to the hepatocyte lysates (70 μg) and incubated at−80 °C overnight. Samples were centrifuged (13,000 *x g*, 20 min) and the protein pellets were dried in a vacuum concentrator and resuspended in freshly prepared 8 M urea in 100 mM ammonium bicarbonate (AmBic) to a concentration of approximately 1 mg/mL. The proteins were reduced using dithiothreitol (DTT, 4.5 mM in 100 mM AmBic) for 1 hour at 37 °C and subsequently alkylated at room temperature in the dark for 30 min with iodoacetamide (10.0 mM in 100 mM AmBic). Excess iodoacetamide was quenched with DTT/100 mM AmBic and the final urea concentration was reduced to 4 M using 100 mM AmBic.

Protein digestion utilized a two-step procedure [83]. Lys-C was added at a ratio of 1:50 (Lys-C:protein) and incubated overnight at 37 °C with shaking. After dilution with 100 mM AmBic to yield a final urea concentration of 1.5 M, trypsin was added at the same ratio. Samples were incubated for 4 hours at 37 °C with shaking. Digestion was quenched by adding trifluoroacetic acid (TFA) until the pH was less than 3.0. The resultant acidified peptides were desalted using C_18_ OMIX Tips (Agilent Technologies, Santa Clara, CA). The tips were conditioned prior to use with LC-MS grade methanol and then subsequently equilibrated with solvent 1 (50:50 water:acetonitrile supplemented with 0.1% (v/v) TFA) followed by solvent 2 (98:2 water:acetonitrile supplemented with 0.1% (v/v) TFA). The peptides were bound to the tips by repeated solution aspiration and dispensing, desalted using solvent 2, and eluted using solvent 1. The recovered peptides were dried in a vacuum concentrator and reconstituted in solvent 2 (1 μg/μL) for analysis via liquid chromatography and tandem mass spectrometry (LC-MS/MS).

### PEM isolation and processing

The PEMs were removed from each well and treated with trypsin (0.25% EDTA) for seven min to remove any bound cells and rinsed in 0.1X PBS (pH = 7.4). PEMs were subsequently snap frozen in liquid nitrogen and stored at−80 °C until further processing. PEMs from the 3D models and unused PEMs, all of identical surface area, were suspended in freshly prepared 8 M urea in 100 mM AmBic and heated at 95 °C overnight. PEMs were subsequently subjected to the same reduction, alkylation, digestion, and desalting steps as described in the previous section.

### LC-MS analysis

Approximately 3 μg of each peptide sample was loaded onto an UPLC (Acquity I-class, Waters Corp.) equipped with a CSH130 C_18_ 1.7 μm, 1.0 mm x 150 mm column maintained at 45 °C. The mobile phases were 0.1% (v/v) formic acid in water (Solvent A) and 0.1% (v/v) formic acid in acetonitrile (Solvent B). Separations were performed at a flow rate of 50 μL/min, using a 110-min gradient from 3–40% solvent B, with all samples analyzed in duplicate. The column effluent was sprayed directly into a Synapt G2-S mass spectrometer using the high definition mass spectrometry (HDMS^E^) mode (continuum, positive-ion, “resolution” MS settings with ion mobility separation of peptides prior to fragmentation). Source conditions were as follows: capillary voltage, 3.0 kV; source temperature, 120 °C; sampling cone, 60 V; desolvation temperature, 350 °C; cone gas flow, 50 L/hr; desolvation gas flow, 500 L/hr; nebulizer gas flow, 6 bar. Both low energy (4 and 2 V in the trap and transfer regions, respectively) and elevated energy (4 V in the trap and ramped from 20 to 50 V in the transfer region) scans were 1.2 seconds each for the m/z range of 50 to 1800. The ion mobility separation (IMS) and transfer wave velocities were 600 and 1200 m/sec, respectively. Wave height within the ion mobility cell was ramped from 10 to 40 V. For lock mass correction, a 1.2 sec low energy scan was acquired every 30 sec from a 100 fmol/μL [Glu1]-fibrinopeptide B solution (50:50 acetonitrile:water supplemented with 0.1% (v/v) formic acid). The infusion rate of the lock spray was 10 μL/min, and introduced into the mass spectrometer at a capillary voltage of 3.0 kV. Lock mass correction was invoked during the data analysis phase of the work.

### Data analysis

Raw data files were analyzed using Protein Lynx Global Server (PLGS, Version. 3.0, Waters Corporation, Milford, MA). LC-MS/MS data was queried against a combined rat and bovine proteome concatenated with a randomized decoy database, allowing for two missed trypsin cleavages. A minimum peptide length was set to five amino acids, with carbamidomethylation of cysteine set as a fixed modification. Oxidation of methionine, proline (i.e., hydroxyproline) and lysine (i.e. hydroxylysine) were set as variable modifications. Additionally, galactosylation and glucosylgalactosylation of lysine, glycosylation of proline, conversion of *N*-terminal glutamine to pyro-glutamate and deamidation of asparagine and glutamine were also set as variable modifications. The criteria used for protein identification utilized both a false discovery rate (FDR) of less than 5% and required at least two peptides per protein. The raw PLGS output was then processed using ISOQuant’s Top3 method [45], where the summed intensities of the three most intense peptides was compared across culture conditions, employing a FDR of less than 1%. Proteins abundances that changed significantly were then analyzed manually and computationally via the DAVID interface [50] to assess enriched Kyoto Encyclopedia of gene and genomes (KEGG) pathways. Protein identification for the PEMs was performed in the same way with the addition of the following variable modifications: allysine and hydroxyallysine, and carbamylation of both lysine and the amino terminus. The database used contained the contaminants list and the rat proteome limited to those associated with the ECM (392 proteins) [84]. This database was derived using the following criteria in UniProt: Gene ontology (GO) term, go:0031012, which corresponds to the extracellular matrix and both the organism terms, *Rattus norvegicus* (Rat) [10116] and proteome:up000002494. This database also contained randomized decoy sequence to allow for determination of a false discovery rate. The mass spectrometry proteomics data for the hepatocyte lysates have been deposited to the ProteomeXchange Consortium [85] via the PRIDE partner repository with the dataset identifier PXD002491.

### Ketone body measurements

LC-MS grade methanol (800 μL) was added to spent cell culture media (200 μL) and incubated at−80 °C overnight. Centrifugation (13,000 *x g*, 20 min) was subsequently performed and the supernatant was collected for LC-MS/MS analysis. Samples (5 μL) were injected into a 3200 QTrap LC-MS/MS (AB Sciex, Framingham, MA) utilizing an Agilent 1100 series high performance liquid chromatography (HPLC) and autosampler (Agilent Technologies, Santa Clara, CA), which was operated in negative ion mode. Separations were accomplished via hydrophilic interaction liquid chromatography (HILIC; Kinetex 2.6 μm, 100 Å, 100 x 2.1 mm, Phenomenex, Torrence, CA). The column was equilibrated using 100% mobile phase A (95:5, acetonitrile:50 mM ammonium formate, pH 3.2) for 20 min. Gradient conditions were employed as followed: 0–8 min, 100% A; 8–16 min, linear gradient up to 30% B (50:40:10 acetonitrile:water:ammonium formate, pH = 3.2); 16–18 min, linear gradient up to 100% B; 18–24 min, 100% B; 24–34 min, linear gradient to 100% A. The column was then re-equilibrated for 20 min with mobile phase A before the injection of the next sample.

MRM was used in negative ion mode to detect the precursor/product ion pairs of 103.1/58.9 and 101.1/56.9 for β-hydroxybutyrate and acetoacetate, respectively. The mass spectrometer acquisition parameters were set as follows: source temperature 300 °C; ion spray voltage,−4000.0 V; declustering potential,−17.0 V, entrance potential,−7.0 V, collision energy,−30.0 V, and collision cell exit potential,−5.0 V. The results were quantified by comparing the peak areas of known concentrations of β-hydroxybutyrate and acetoacetate in Analyst (v.1.6, AB Sciex, Framingham, MA). Concentrations used for quantification ranged from 1 μM–60 μM, prepared in fresh unused hepatocyte media.

### Albumin, urea and glucose measurements

Albumin concentrations in spent culture media were measured via an enzyme linked immunosorbent assay (ELISA) using a polyclonal antibody against rat albumin. Urea concentrations were measured using a BUN assay kit according to the manufacturer’s protocol (Stanbio Laboratory, Boerne, TX). Standard curves were generated using purified rat albumin and urea diluted in fresh hepatocyte media and levels were normalized to total protein content. Glucose concentrations were determined with a glucose analyzer (YSI 2700, Yellow Springs Instruments, Yellow Springs, OH), maintained at the Virginia Tech Metabolic Phenotyping Core Facility.

### Statistical analyses

All data are reported as mean ± standard deviation and are *n* = 3 unless otherwise specified. Statistical significance was determined using a two-tailed Student’s *t*-test. The Benjamini-Hochberg correction was applied to account for multiple hypotheses testing with corrected *p* values (*p* < 0.05) being considered significant.

## Additional file

Additional file 1: Supporting Figures and Tables. Protein list for the pairwise comparison of 3DHL vs. 3DH. The original figure from the manuscript is shown (Fig. 3a). Protein descriptions are as provided by the UniProt ID, with the exception of those with an asterisk (*). These proteins were manually annotated by a BLAST search of the originally identified protein. For example, D3ZFA8 was computationally identified as “Protein LOC100362366 OS = Rattus norvegicus GN = LOC100364909 PE = 3 SV = 1”. This sequence was used in a BLAST search to identify identical proteins (>95%) in the rat or murine database. The protein description for that protein was then inserted but keeping the original UniProt ID. **Figure S1B.** Protein list for the pairwise comparison of 3DHL vs. HM. The original figure from the manuscript is shown (Fig. 3b). Protein UniProt Accessions with an asterisk (*) were manually annotated using the results of a BLAST search of the originally identified protein (See **Figure S1A.** for details). **Table S1.** Pearson Coefficients Across Model Configurations and Replicates. The first column and row indicate individual LC-MS runs organized by replicates. Biological replicates are separate constructs (in triplicate) and technical replicates are duplicate injections of the same sample (see Key). Averaged Pearson Correlations between constructs and replicates are shown to the right of the Key. **Table S2.** DAVID Analysis of Enriched KEGG pathways. Terms exhibiting *p*-values less than 0.05 are shown. Arranged alphabetically according to the KEGG pathway. Multiple entries are for separate pairwise comparisons. *p*-values < 0.0001 (1.0E-04) are shown in bold. Pathways with over 200 proteins were excluded from the list. **Figure S2.** Peroxisomal processes are modulated by PEMs and LSECs. Relative protein abundance ratios based on the Top3 ion intensities for enzymes associated with peroxisomal fatty acid β-oxidation that change significantly based upon the culture conditions (* *p* < 0.05 as compared to HMs). (PDF 3570 kb)

## Abbreviations

AmBic: Ammonium bicarbonate
CS: Collagen sandwich
DIA: Data-independent acquisition
ECM: Extracellular matrix
FDR: False discovery rate
HA: Hyaluronic acid
HM: Hepatocyte monolayer
LSECs: Liver sinusoidal endothelial cells
MRM: Multiple reaction monitoring
PEM: Polyelectrolyte multilayer
SoD: Space of Disse
