# Supplementary material for: The hepatocyte proteome in organotypic rat liver models and the influence of the local microenvironment
Source: Proteome Sci. 2017 Jun 20;15:12. doi: 10.1186/s12953-017-0120-6 (PMC5480101; doi:10.1186/s12953-017-0120-6)
Supplement: Additional file 1: Supporting Figures and Tables. — Protein list for the pairwise comparison of 3DHL vs. 3DH. The original figure from the manuscript is shown (Fig. 3a). Protein descriptions are as provided by the UniProt ID, with the exception of those with an asterisk (*). These proteins were manually annotated by a BLAST search of the originally identified protein. For example, D3ZFA8 was computationally identified as “Protein LOC100362366 OS = Rattus norvegicus GN = LOC100364909 PE = 3 SV = 1”. This sequence was used in a BLAST search to identify identical proteins (>95%) in the rat or murine database. The protein description for that protein was then inserted but keeping the original UniProt ID. Figure S1B. Protein list for the pairwise comparison of 3DHL vs. HM. The original figure from the manuscript is shown (Fig. 3b). Protein UniProt Accessions with an asterisk (*) were manually annotated using the results of a BLAST search of the originally identified protein (See Figure S1A. for details). Table S1. Pearson Coefficients Across Model Configurations and Replicates. The first column and row indicate individual LC-MS runs organized by replicates. Biological replicates are separate constructs (in triplicate) and technical replicates are duplicate injections of the same sample (see Key). Averaged Pearson Correlations between constructs and replicates are shown to the right of the Key. Table S2. DAVID Analysis of Enriched KEGG pathways. Terms exhibiting p-values less than 0.05 are shown. Arranged alphabetically according to the KEGG pathway. Multiple entries are for separate pairwise comparisons. p-values < 0.0001 (1.0E-04) are shown in bold. Pathways with over 200 proteins were excluded from the list. Figure S2. Peroxisomal processes are modulated by PEMs and LSECs. Relative protein abundance ratios based on the Top3 ion intensities for enzymes associated with peroxisomal fatty acid β-oxidation that change significantly based upon the culture conditions (* p < 0.05 as compared to HMs). (PDF 3570 kb) [file 12953_2017_120_MOESM1_ESM.pdf]

SUPPLEMENTAL MATERIALS

The Hepatocyte Proteome in Organotypic Rat Liver Models and the Influence of the Local Microenvironment

Lucas T. Vu[1], Sophia M. Orbach[1], W. Keith Ray[2], Margaret E. Cassin[1], Padmavathy Rajagopalan[1,3,4], Richard F. Helm[2,4*]

[1]Department of Chemical Engineering, Virginia Tech, Blacksburg, VA
[2]Department of Biochemistry, Virginia Tech, Blacksburg, VA
[3]School of Biomedical Engineering and Sciences, Virginia Tech, Blacksburg, VA
[4]ICTAS Center for Systems Biology and Engineered Tissues, Virginia Tech, Blacksburg, VA

Corresponding Author: Richard F. Helm (helmrf@vt.edu)

Table of Contents

**Figure S1**	Protein lists for the pairwise comparison of the three constructs. (A) 3DHL *vs.* 3DH, (B) 3DHL *vs.* HM, (C) 3DH *vs.* HM.	2
**Table S1**	Sample to Sample Pearson Correlations for Technical and Biological Replicates.	5
**Table S2**	DAVID Analysis of Enriched KEGG pathways.	6
**Figure S2**	Peroxisomal processes are modulated by PEMs and LSECs.	8

Hepatocyte Proteome Data: ProteomeXchange Consortium via the PRIDE partner repository with the dataset identifier PXD002491.

Website: https://www.ebi.ac.uk/pride/archive/login

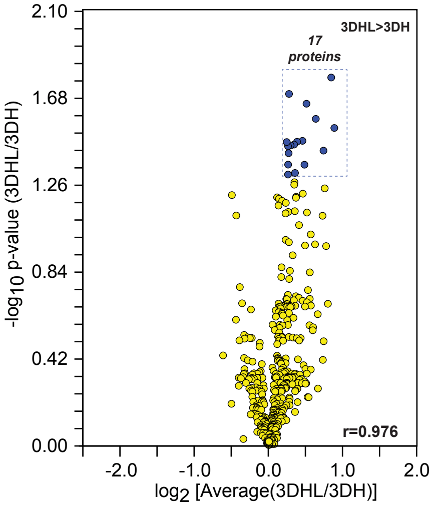

Protein Description	UniProt ID
40S ribosomal protein S17, GN=Rps17	D3ZFA8*
78 kDa glucose-regulated protein GN=Hspa5	P06761
Calnexin GN=Canx	P35565
Carboxylesterase 1D GN=Ces1d	P16303
Carboxylesterase 4 (Liver) (EST4_RAT)	Q64573
Carboxylesterase B-1 (Liver) (EST5_RAT)	Q63010
Cytochrome P450 2A1 GN=Cyp2a1	P11711
Cytochrome P450 2C12, female-specific GN=LOC100361492	Q64648
Endoplasmic reticulum resident protein 29 GN=Erp29	P52555
Glycine N-acyltransferase GN=Glyat	Q5PQT3
Peptidyl-prolyl cis-trans isomerase B GN=Ppib	P24368
Protein disulfide-isomerase A4 GN=Pdia4	P38659
Protein disulfide-isomerase GN=P4hb	P04785
Rab GDP dissociation inhibitor beta GN=Gdi2	P50399
Thioredoxin GN=Txn	P11232
UDP-glucuronosyltransferase 1-6 GN=Ugt1a6	P08430
Voltage-dependent anion-selective channel protein 1 GN=Vdac1	Q9Z2L0

Fig S1A. Protein list for the pairwise comparison of 3DHL vs. 3DH. The original figure from the manuscript is shown (Fig. 3A). Protein descriptions are as provided by the UniProt ID, with the exception of those with an asterisk (*). These proteins were manually annotated by a BLAST search of the originally identified protein. For example, D3ZFA8 was computationally identified as “Protein LOC100362366 OS=Rattus norvegicus GN=LOC100364909 PE=3 SV=1”. This sequence was used in a BLAST search to identify identical proteins (>95 %) in the rat or murine database. The protein description for that protein was then inserted but keeping the original UniProt ID.

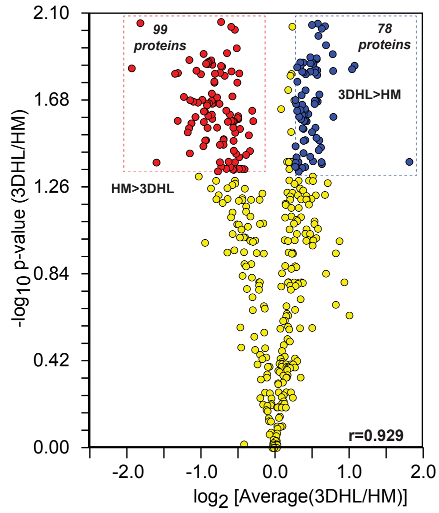

Fig S1B. Protein list for the pairwise comparison of 3DHL vs. HM. The original figure from the manuscript is shown (Fig. 3B). Protein UniProt Accessions with an asterisk (*) were manually annotated using the results of a BLAST search of the originally identified protein (See Fig. S1A for details).

3DHL abundance > HM (Blue)

Protein Description	UniProt ID
10-formyltetrahydrofolate dehydrogenase GN=Aldh1l2	D3ZTP0
2,4-dienoyl-CoA reductase, mitochondrial GN=Decr1	Q64591
3-ketoacyl-CoA thiolase A, peroxisomal GN=Acaa1a	P21775
3-ketoacyl-CoA thiolase, mitochondrial GN=Acaa2	G3V9U2
3-oxo-5-alpha-steroid 4-dehydrogenase 1 (Fragment) GN=Srd5a1	G8JLS2
4-nitrophenylphosphatase domain/non-neuronal SNAP25-like protein homolog 1 GN=Nipsnap1	G3V728
Acyl-CoA synthetase family member 2, mitochondrial GN=Acsf2	Q499N5
Acyl-CoA synthetase short-chain family member 3, mitochondrial GN=Acss3	D3ZGU2
Acyl-Coenzyme A dehydrogenase, very long chain GN=Acadvl	Q5M9H2
Acyl-coenzyme A oxidase GN=Acox1	F1LQC1
Acyl-coenzyme A synthetase ACSM5, mitochondrial GN=Acsm5	Q6AYT9
Adipocyte plasma membrane-associated protein GN=Apmap	Q7TP48
Agmatinase, mitochondrial GN=Agmat	Q0D2L3
Aldehyde dehydrogenase family 6, subfamily A1, isoform CRA_b GN=Aldh6a1	G3V7J0
Aldehyde dehydrogenase X, mitochondrial GN=Aldh1b1	G3V7I5
Alpha-aminoadipic semialdehyde dehydrogenase GN=Aldh7a1	Q64057
Alpha-aminoadipic semialdehyde synthase, mitochondrial GN=Aass	D4ACE9
Alpha-methylacyl-CoA racemase GN=Amacr	P70473
ATP synthase subunit O, mitochondrial GN=Atp5o	Q06647
Bile acid-CoA:amino acid N-acyltransferase GN=Baat	Q63276
Biphenyl hydrolase-like (Serine hydrolase) GN=Bphl	Q3B8N9
Carboxylesterase 1D GN=Ces1d	P16303
Carboxylesterase 1E GN=Ces1e	Q63108
Catalase GN=Cat	P04762
Choline dehydrogenase, mitochondrial GN=Chdh	Q6UPE0
Dihydrolipoamide S-succinyltransferase (E2 component of 2-oxo-glutarate complex) GN=Dlst	G3V6P2
Dihydrolipoyl dehydrogenase, mitochondrial GN=Dld	Q6P6R2
Dimethylglycine dehydrogenase, mitochondrial GN=Dmgdh	Q63342
Dolichyl-diphosphooligosaccharide--protein glycosyltransferase 48 kDa subunit GN=Ddost	F1LM69
Dolichyl-diphosphooligosaccharide--protein glycosyltransferase subunit 1 GN=Rpn1	Q6P7A7
Ectonucleoside triphosphate diphosphohydrolase 5 GN=Entpd5	Q6P6S9
Electron transfer flavoprotein subunit alpha, mitochondrial GN=Etfa	P13803
Electron transfer flavoprotein subunit beta GN=Etfb	Q68FU3
Elongation factor Tu, mitochondrial GN=Tufm	P85834
Enoyl-CoA delta isomerase 1, mitochondrial GN=Eci1	P23965
Enoyl-CoA delta isomerase 2, mitochondrial GN=Eci2	Q5XIC0
Enoyl-CoA hydratase domain-containing protein 3, mitochondrial GN=Echdc3	Q3MIE0
Enoyl-CoA hydratase, mitochondrial GN=Echs1	P14604
Fumarylacetoacetate hydrolase domain-containing protein 2 GN=Fahd2	B2RYW9
Glutamate dehydrogenase 1, mitochondrial GN=Glud1	P10860
Glutaryl-Coenzyme A dehydrogenase (Predicted) GN=Gcdh	D3ZT90
Glutathione S-transferase kappa 1 GN=Gstk1	P24473
Glycine N-acyltransferase GN=Glyat	Q5PQT3
GTP:AMP phosphotransferase AK4, mitochondrial GN=Ak4	Q9WUS0
Haloacid dehalogenase-like hydrolase domain containing 3 GN=Hdhd3	B2RYT7
Hydroxyacid oxidase 1 GN=Hao1	B0BNF9
Hydroxyacyl-coenzyme A dehydrogenase, mitochondrial GN=Hadh	Q9WVK7
Hydroxymethylglutaryl-CoA lyase, mitochondrial GN=Hmgcl	P97519
Isovaleryl-CoA dehydrogenase, mitochondrial GN=Ivd	P12007
Kynurenine--oxoglutarate transaminase 3 GN=Ccbl2	Q58FK9
Kynurenine/alpha-aminoadipate aminotransferase, mitochondrial GN=Aadat	Q64602
Liver carboxylesterase 4	Q64573
Liver carboxylesterase B-1	Q63010
Long-chain-fatty-acid--CoA ligase 5 GN=Acsl5	O88813
Methylcrotonoyl-CoA carboxylase beta chain, mitochondrial GN=Mccc2	Q5XIT9
Microsomal triglyceride transfer protein GN=Mttp	D4A1W8
MOSC domain-containing protein 2, mitochondrial GN=Marc2	D3Z900
NADH dehydrogenase [ubiquinone] 1 alpha subcomplex subunit 9, mitochondrial GN=Ndufa9	Q5BK63
NADH dehydrogenase [ubiquinone] iron-sulfur protein 2, mitochondrial GN=Ndufs2	Q641Y2
Non-specific lipid-transfer protein GN=Scp2	P11915
Ornithine aminotransferase, mitochondrial GN=Oat	P04182
Peroxiredoxin-4 GN=Prdx4	Q9Z0V5
Peroxisomal bifunctional enzyme GN=Ehhadh	P07896
Peroxisomal multifunctional enzyme type 2 GN=Hsd17b4	P97852
Probable 2-oxoglutarate dehydrogenase E1 component DHKTD1, mitochondrial GN=Dhtkd1	Q4KLP0
Protein disulfide-isomerase A6 GN=Pdia6	Q63081
Sarcosine dehydrogenase, mitochondrial GN=Sardh	Q64380
Short/branched chain specific acyl-CoA dehydrogenase, mitochondrial GN=Acadsb	P70584
Succinate dehydrogenase [ubiquinone] iron-sulfur subunit, mitochondrial GN=Sdhb	P21913
Succinyl-CoA ligase subunit beta GN=Sucla2	F1LM47
Succinyl-CoA ligase subunit beta GN=Suclg2	F1LPV8
Sulfated glycoprotein 1 GN=Psap	P10960
Taste receptor type 1 member 2 GN=Tas1r2	F8WFI0
Trifunctional enzyme subunit alpha, mitochondrial GN=Hadha	Q64428
Trifunctional enzyme subunit beta, mitochondrial GN=Hadhb	Q60587
UDP-glucuronosyltransferase 1-6 GN=Ugt1a6	P08430
UDP-glucuronosyltransferase 2B15 GN=Ugt2b15	P36511
UDP-glucuronosyltransferase Ugt2b10 GN=Ugt2b10	D4A132

HM abundance > 3DHL (Red)

Protein Description	UniProt ID
14-3-3 protein zeta/delta GN=Ywhaz	P63102
40S ribosomal protein S12 GN=LOC100360573	D3ZHB3
40S ribosomal protein S15 GN=Rps15	P62845
40S ribosomal protein S18 (Fragment) GN=LOC100912024	M0R5K9*
40S ribosomal protein S19 GN=LOC100362339	D4A6G6*
40S ribosomal protein S24 GN=LOC100363469	D4ACJ1
40S ribosomal protein S25 (Fragment) GN=RGD1564597	F1M6F4*
40S ribosomal protein S28 GN=Rps28	P62859
40S ribosomal protein S3 GN=Rps3	P62909
40S ribosomal protein S3a GN=LOC100365839	M0R6L4
40S ribosomal protein S4, X isoform GN=Rps4x	P62703
40S ribosomal protein S7 GN=Rps7	P62083
40S ribosomal protein S8 GN=Rps8	P62243
40S ribosomal protein SA GN=Rpsa	P38983
6-phosphogluconate dehydrogenase, decarboxylating GN=Pgd	P85968
60S acidic ribosomal protein P2 GN=LOC498555	D4A4D5*
60S ribosomal protein L11 GN=Rpl11	P62914
60S ribosomal protein L12 GN=Rpl12	P23358
60S ribosomal protein L13 GN=LOC100360491	D3ZRM9
60S ribosomal protein L17 GN=Rpl17	P24049
60S ribosomal protein L18 GN=Rpl18	P12001
60S ribosomal protein L23 GN=Rpl23	P62832
60S ribosomal protein L23a GN=LOC689899	D3ZTH8*
60S ribosomal protein L24 GN=Rpl24	P83732
60S ribosomal protein L4 GN=Rpl4	Q6P3V9
60S ribosomal protein L6 GN=Rpl6	H7C5Y5
60S ribosomal protein L7 GN=Rpl7	P05426
60S ribosomal protein L7a (Fragment) GN=LOC100910109	F1M013*
60S ribosomal protein L9 (Fragment) GN=RGD1561928	F1M7A9*
Actin, alpha cardiac muscle 1 GN=Actc1	P68035
Actin, cytoplasmic 1 GN=Actb	P60711
Adenosylhomocysteinase GN=Ahcy	P10760
Alcohol dehydrogenase [NADP(+)] GN=Akr1a1	P51635
Alcohol dehydrogenase 1 GN=Adh1	P06757
Alpha-enolase GN=Eno1	P04764
Annexin A2 GN=Anxa2	Q07936
Annexin A5 GN=Anxa5	P14668
Apolipoprotein E GN=Apoe	P02650
Apoptosis-inducing factor 1, mitochondrial GN=Aifm1	Q9JM53
Arginase-1 GN=Arg1	P07824
Argininosuccinate synthase GN=Ass1	P09034
Betaine--homocysteine S-methyltransferase 1 GN=Bhmt	O09171
Calmodulin GN=Calm1	P62161
CArG-binding factor A GN=Hnrnpab	Q9QX80
Catechol O-methyltransferase GN=Comt	P22734
Cathepsin B GN=Ctsb	P00787
Chaperonin containing Tcp1, subunit 6A (Zeta 1) GN=Cct6a	Q3MHS9
Cofilin-1 GN=Cfl1	P45592
Cytosol aminopeptidase GN=Lap3	Q68FS4
D-dopachrome decarboxylase GN=Ddt	P80254
Elongation factor 1-alpha 1 GN=Eef1a1	P62630
Elongation factor 2 GN=Eef2	P05197
Eukaryotic translation elongation factor 1 beta 2 GN=Eef1b2	B5DEN5
Filamin alpha GN=Flna	C0JPT7
Fructose-1,6-bisphosphatase 1 GN=Fbp1	P19112
Fructose-bisphosphate aldolase GN=Aldob	Q66HT1
Fructose-bisphosphate aldolase A GN=Aldoa	P05065
Fumarylacetoacetase GN=Fah	F1M6W1
Glucose-6-phosphate isomerase GN=Gpi	Q6P6V0
Glutathione S-transferase Mu 1 GN=Gstm1	P04905
Glutathione S-transferase Mu 2 GN=Gstm2	P08010
Glycerol-3-phosphate dehydrogenase [NAD(+)], cytoplasmic GN=Gpd1	O35077
Heat shock protein HSP 90-beta GN=Hsp90ab1	P34058
Heterogeneous nuclear ribonucleoprotein A3 GN=Hnrnpa3	Q6URK4
Heterogeneous nuclear ribonucleoproteins A2/B1 GN=Hnrnpa2b1	F1LM82
Histone H1.4 GN=Hist1h1e	P15865
Histone H3 GN=RGD1564447	D3ZK97
Keratin, type I cytoskeletal 18 GN=Krt18	Q5BJY9
Keratin, type II cytoskeletal 8 GN=Krt8	Q10758
Leucine-rich repeat-containing protein 59 GN=Lrrc59	Q5RJR8
Malate dehydrogenase, cytoplasmic GN=Mdh1	O88989
Myosin light polypeptide 6 GN=Myl6	Q64119
Myosin regulatory light chain RLC-A GN=Rlc-a	P13832
Myosin-14 GN=Myh14	F1LNF0
Myosin, heavy polypeptide 9, non-muscle GN=LOC100911597	G3V6P7
Nucleophosmin GN=Npm1	P13084
Nucleoside diphosphate kinase B GN=Nme2	P19804
Peptidyl-prolyl cis-trans isomerase A GN=Ppia	P10111
Peroxiredoxin-6 GN=Prdx6	O35244
Peroxisomal trans-2-enoyl-CoA reductase GN=Pecr	Q9WVK3
Phosphate carrier protein, mitochondrial GN=Slc25a3	G3V741
Phosphatidylethanolamine-binding protein 1 GN=Pebp1	P31044
Phosphoglycerate kinase 1 GN=Pgk1	P16617
Profilin-1 GN=Pfn1	P62963
Prostaglandin reductase 1 GN=Ptgr1	P97584
Pyruvate kinase isozymes R/L GN=Pklr	P12928
Rab GDP dissociation inhibitor beta GN=Gdi2	P50399
S-adenosylmethionine synthase isoform type-1 GN=Mat1a	P13444
Selenium-binding protein 1 (Fragment) GN=Selenbp1	F1LRJ9
Serine/arginine-rich splicing factor 3 GN=Srsf3	Q0ZFS8
Serum albumin GN=Alb	P02770
T-complex protein 1 subunit gamma GN=Cct3	Q6P502
Thioredoxin GN=Txn	P11232
Transgelin-2 GN=Tagln2	Q5XFX0
Transitional endoplasmic reticulum ATPase GN=Vcp	P46462
Transketolase GN=Tkt	P50137
Triosephosphate isomerase GN=Tpi1	P48500
Tropomyosin alpha-3 chain GN=Tpm3	Q63610
Tropomyosin alpha-4 chain GN=Tpm4	P09495

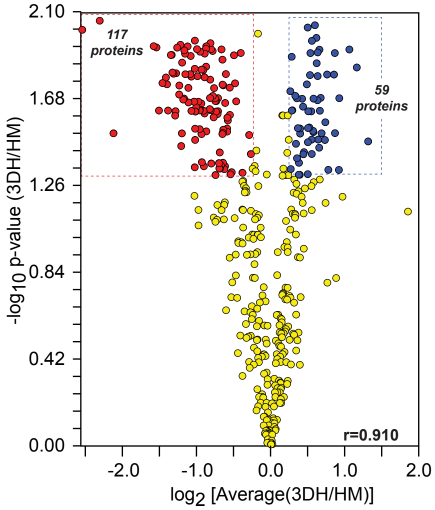

3DH protein abundance > HM (Blue)

Protein Description	UniProt ID
10-formyltetrahydrofolate dehydrogenase GN=Aldh1l2	D3ZTP0
2,4-dienoyl-CoA reductase, mitochondrial GN=Decr1	Q64591
3-hydroxyacyl-CoA dehydrogenase type-2 GN=Hsd17b10	B0BMW2
3-ketoacyl-CoA thiolase A, peroxisomal GN=Acaa1a	P21775
4-aminobutyrate aminotransferase, mitochondrial GN=Abat	P50554
4-nitrophenylphosphatase domain/non-neuronal SNAP25-like protein homolog 1 GN=Nipsnap1	G3V728
60 kDa heat shock protein, mitochondrial GN=Hspd1	P63039
Acyl-CoA dehydrogenase family member 11 GN=Acad11	B3DMA2
Acyl-CoA synthetase family member 2, mitochondrial GN=Acsf2	Q499N5
Acyl-coenzyme A oxidase GN=Acox1	F1LQC1
Acyl-coenzyme A oxidase GN=Acox3	F1M9A7
Acyl-coenzyme A synthetase ACSM5, mitochondrial GN=Acsm5	Q6AYT9
Adipocyte plasma membrane-associated protein GN=Apmap	Q7TP48
Agmatinase, mitochondrial GN=Agmat	Q0D2L3
Aldehyde dehydrogenase family 6, subfamily A1 GN=Aldh6a1	G3V7J0
Aldehyde dehydrogenase X, mitochondrial GN=Aldh1b1	G3V7I5
Alpha-aminoadipic semialdehyde dehydrogenase GN=Aldh7a1	Q64057
Alpha-aminoadipic semialdehyde synthase, mitochondrial GN=Aass	D4ACE9
ATP synthase subunit O, mitochondrial GN=Atp5o	Q06647
Bile acid-CoA:amino acid N-acyltransferase GN=Baat	Q63276
Carboxylesterase 1E GN=Ces1e	Q63108
Catalase GN=Cat	P04762
D-beta-hydroxybutyrate dehydrogenase, mitochondrial GN=Bdh1	P29147
Dimethylaniline monooxygenase [N-oxide-forming] 5 GN=Fmo5	Q8K4C0
Dimethylglycine dehydrogenase, mitochondrial GN=Dmgdh	Q63342
Electron transfer flavoprotein subunit alpha, mitochondrial GN=Etfa	P13803
Electron transfer flavoprotein subunit beta GN=Etfb	Q68FU3
Elongation factor Tu, mitochondrial GN=Tufm	P85834
Enoyl-CoA delta isomerase 2, mitochondrial GN=Eci2	Q5XIC0
Ethylmalonic encephalopathy 1 GN=Ethe1	B0BNJ4
Fatty acid synthase GN=Fasn	P12785
Glucosidase 2 subunit beta GN=Prkcsh	B1WC34
Glutaryl-Coenzyme A dehydrogenase (Predicted) GN=Gcdh	D3ZT90
Glutathione S-transferase kappa 1 GN=Gstk1	P24473
GTP:AMP phosphotransferase AK4, mitochondrial GN=Ak4	Q9WUS0
Haloacid dehalogenase-like hydrolase domain containing 3 GN=Hdhd3	B2RYT7
Heterogeneous nuclear ribonucleoprotein K GN=Hnrnpk	P61980
Hydroxyacid oxidase 1 GN=Hao1	B0BNF9
Isovaleryl-CoA dehydrogenase, mitochondrial GN=Ivd	P12007
Kynurenine/alpha-aminoadipate aminotransferase, mitochondrial GN=Aadat	Q64602
Long-chain-fatty-acid--CoA ligase 5 GN=Acsl5	O88813
NADH dehydrogenase [ubiquinone] 1 a-subcomplex subunit 9, mitochondrial GN=Ndufa9	Q5BK63
Nicotinamide nucleotide transhydrogenase GN=Nnt	Q5BJZ3
Non-specific lipid-transfer protein GN=Scp2	P11915
Ornithine aminotransferase, mitochondrial GN=Oat	P04182
Peroxisomal bifunctional enzyme GN=Ehhadh	P07896
Peroxisomal multifunctional enzyme type 2 GN=Hsd17b4	P97852
Probable 2-oxoglutarate dehydrogenase E1 component DHKTD1, mitochondrial GN=Dhtkd1	Q4KLP0
Sarcosine dehydrogenase, mitochondrial GN=Sardh	Q64380
Serine hydroxymethyltransferase GN=Shmt2	Q5U3Z7
Short-chain specific acyl-CoA dehydrogenase, mitochondrial GN=Acads	P15651
Short/branched chain specific acyl-CoA dehydrogenase, mitochondrial GN=Acadsb	P70584
Sterol 26-hydroxylase, mitochondrial GN=Cyp27a1	P17178
Succinyl-CoA ligase subunit beta GN=Sucla2	F1LM47
Taste receptor type 1 member 2 GN=Tas1r2	F8WFI0
Trifunctional enzyme subunit beta, mitochondrial GN=Hadhb	Q60587
UDP-glucuronosyltransferase 2B15 GN=Ugt2b15	P36511
UDP-glucuronosyltransferase 2B2 GN=Ugt2b15	F1LM22
Very long-chain acyl-CoA synthetase GN=Slc27a2	P97524

Fig S1C. Protein list for the pairwise comparison of 3DH vs. HM. The original figure from the manuscript is shown (Fig. 3C). Protein UniProt Accessions with an asterisk (*) were manually annotated by a BLAST search of the originally identified protein (See Fig. S1A for details).

HM abundance > 3DH (Red)

Protein Description	UniProt ID
14-3-3 protein zeta/delta GN=Ywhaz	P63102
2-oxoglutarate dehydrogenase, mitochondrial GN=Ogdh	Q5XI78
3-alpha-hydroxysteroid dehydrogenase GN=Akr1c9	P23457
40S ribosomal protein S12 GN=LOC100360573	D3ZHB3
40S ribosomal protein S15 GN=Rps15	P62845
40S ribosomal protein S17 GN=LOC100364909	D3ZFA8*
40S ribosomal protein S19 GN=LOC100362339	D4A6G6*
40S ribosomal protein S24 GN=LOC100363469	D4ACJ1
40S ribosomal protein S25 (Fragment) GN=RGD1564597	F1M6F4*
40S ribosomal protein S28 GN=Rps28	P62859
40S ribosomal protein S3 GN=Rps3	P62909
40S ribosomal protein S4, X isoform GN=Rps4x	P62703
40S ribosomal protein S6 (Fragment) GN=Rps6	M0RD75
40S ribosomal protein S7 GN=Rps7	P62083
40S ribosomal protein S8 GN=Rps8	P62243
40S ribosomal protein SA GN=Rpsa	P38983
6-phosphogluconate dehydrogenase, decarboxylating GN=Pgd	P85968
60S acidic ribosomal protein P2 GN=LOC498555	D4A4D5*
60S ribosomal protein L11 GN=Rpl11	P62914
60S ribosomal protein L12 GN=Rpl12	P23358
60S ribosomal protein L13 GN=LOC100360491	D3ZRM9
60S ribosomal protein L17 GN=Rpl17	P24049
60S ribosomal protein L23 GN=Rpl23	P62832
60S ribosomal protein L23a GN=LOC689899	D3ZTH8*
60S ribosomal protein L24 GN=Rpl24	P83732
60S ribosomal protein L6 GN=Rpl6	H7C5Y5
60S ribosomal protein L7a (Fragment) GN=LOC100910109	F1M013*
60S ribosomal protein L9 (Fragment) GN=RGD1561928	F1M7A9*
Ab1-114 GN=Slc25a15	Q7TPA1
Actin, alpha cardiac muscle 1 GN=Actc1	P68035
Actin, cytoplasmic 1 GN=Actb	P60711
Acyl-CoA-binding protein (Fragment) GN=Dbi	M0RDC5
Adenosylhomocysteinase GN=Ahcy	P10760
Alcohol dehydrogenase 1 GN=Adh1	P06757
Aldose reductase-related protein 1 GN=Akr1b7	Q5RJP0
Alpha-enolase GN=Eno1	P04764
Apolipoprotein E GN=Apoe	P02650
Apoptosis-inducing factor 1, mitochondrial GN=Aifm1	Q9JM53
Arginase-1 GN=Arg1	P07824
Argininosuccinate synthase GN=Ass1	P09034
ATP synthase subunit b, mitochondrial GN=Atp5f1	P19511
Betaine--homocysteine S-methyltransferase 1 GN=Bhmt	O09171
Biliverdin reductase B (Flavin reductase (NADPH)) GN=Blvrb	B5DF65
Calmodulin GN=Calm1	P62161
Carboxylesterase 1D GN=Ces1d	P16303
CArG-binding factor A GN=Hnrnpab	Q9QX80
Catechol O-methyltransferase GN=Comt	P22734
Cathepsin B GN=Ctsb	P00787
CDGSH iron-sulfur domain-containing protein 1 GN=Cisd1	B0K020
Chaperonin containing Tcp1, subunit 6A (Zeta 1) GN=Cct6a	Q3MHS9
Cofilin-1 GN=Cfl1	P45592
Cytochrome b-c1 complex subunit 7 GN=Uqcrb	B2RYS2
Cytochrome P450 2C12, female-specific GN=LOC100361492	Q64648
Cytosol aminopeptidase GN=Lap3	Q68FS4
Cytosolic 10-formyltetrahydrofolate dehydrogenase GN=Aldh1l1	P28037
D-dopachrome decarboxylase GN=Ddt	P80254
Destrin GN=Dstn	Q7M0E3
Elongation factor 1-alpha 1 GN=Eef1a1	P62630
Elongation factor 1-delta GN=Eef1d	Q68FR9
Elongation factor 2 GN=Eef2	P05197
Eukaryotic translation elongation factor 1 beta 2 GN=Eef1b2	B5DEN5
Fatty acid-binding protein, liver GN=Fabp1	P02692
Filamin alpha GN=Flna	C0JPT7
Fructose-1,6-bisphosphatase 1 GN=Fbp1	P19112
Fructose-bisphosphate aldolase GN=Aldob	Q66HT1
Fructose-bisphosphate aldolase A GN=Aldoa	P05065
Fumarylacetoacetase GN=Fah	F1M6W1
Glutathione S-transferase alpha-1 GN=Gsta1	P00502
Glutathione S-transferase alpha-2 GN=Gsta2	P04903
Glutathione S-transferase alpha-3 GN=Gsta3	P04904
Glutathione S-transferase Mu 1 GN=Gstm1	P04905
Glutathione S-transferase Mu 2 GN=Gstm2	P08010
Glycerol-3-phosphate dehydrogenase [NAD(+)], cytoplasmic GN=Gpd1	O35077
Heat shock protein HSP 90-alpha GN=Hsp90aa1	P82995
Heterogeneous nuclear ribonucleoprotein A3 GN=Hnrnpa3	Q6URK4
Heterogeneous nuclear ribonucleoproteins A2/B1 GN=Hnrnpa2b1	F1LM82
Hypoxanthine-guanine phosphoribosyltransferase GN=Hprt1	P27605
Keratin, type I cytoskeletal 18 GN=Krt18	Q5BJY9
Keratin, type II cytoskeletal 8 GN=Krt8	Q10758
Leucine-rich repeat-containing protein 59 GN=Lrrc59	Q5RJR8
Macrophage migration inhibitory factor GN=Mif	P30904
Malate dehydrogenase, cytoplasmic GN=Mdh1	O88989
Mitochondrial dicarboxylate carrier GN=Slc25a10	O89035
Myosin regulatory light chain RLC-A GN=Rlc-a	P13832
Myosin-14 GN=Myh14	F1LNF0
NADPH--cytochrome P450 reductase GN=Por	P00388
Nucleophosmin GN=Npm1	P13084
Nucleoside diphosphate kinase B GN=Nme2	P19804
PDZ and LIM domain protein 1 GN=Pdlim1	P52944
Peptidyl-prolyl cis-trans isomerase A GN=Ppia	P10111
Peroxiredoxin-1 GN=Prdx1	Q63716
Peroxiredoxin-5, mitochondrial (Fragment) GN=Prdx5	D3ZEN5
Peroxiredoxin-6 GN=Prdx6	O35244
Phosphate carrier protein, mitochondrial GN=Slc25a3	G3V741
Phosphatidylethanolamine-binding protein 1 GN=Pebp1	P31044
Phosphoglycerate kinase 1 GN=Pgk1	P16617
Profilin-1 GN=Pfn1	P62963
Prostaglandin reductase 1 GN=Ptgr1	P97584
Pyruvate kinase isozymes R/L GN=Pklr	P12928
Rab GDP dissociation inhibitor beta GN=Gdi2	P50399
Regucalcin GN=Rgn	Q03336
Ribonuclease UK114 GN=Hrsp12	P52759
Ribosomal protein S5, isoform CRA_b GN=Rps5	B0BN81
S-adenosylmethionine synthase isoform type-1 GN=Mat1a	P13444
Selenium-binding protein 1 (Fragment) GN=Selenbp1	F1LRJ9
Serum albumin GN=Alb	P02770
Superoxide dismutase [Cu-Zn] GN=Sod1	P07632
T-complex protein 1 subunit beta GN=Cct2	Q5XIM9
T-complex protein 1 subunit gamma GN=Cct3	Q6P502
Thioredoxin GN=Txn	P11232
Transgelin-2 GN=Tagln2	Q5XFX0
Transitional endoplasmic reticulum ATPase GN=Vcp	P46462
Transketolase GN=Tkt	P50137
Triosephosphate isomerase GN=Tpi1	P48500
Tropomyosin alpha-1 chain GN=Tpm1	P04692
Tropomyosin alpha-3 chain GN=Tpm3	Q63610
Tropomyosin alpha-4 chain GN=Tpm4	P09495

	3DHL_1A	3DHL_1B	3DHL_2A	3DHL_2B	3DHL_3A	3DHL_3B	3DH_1A	3DH_1B	3DH_2A	3DH_2B	3DH_3A	3DH_3B	HM_1A	HM_1B	HM_2A	HM_2B	HM_3A	HM_3B
**3DHL_1A**	1.0000	0.9986	0.9787	0.9800	0.9892	0.9928	0.9746	0.9755	0.9742	0.9755	0.9772	0.9774	0.9496	0.9466	0.9354	0.9398	0.9471	0.9424
**3DHL_1B**	0.9986	1.0000	0.9788	0.9795	0.9902	0.9935	0.9760	0.9769	0.9748	0.9756	0.9784	0.9775	0.9481	0.9462	0.9353	0.9391	0.9450	0.9406
**3DHL_2A**	0.9787	0.9788	1.0000	0.9988	0.9813	0.9788	0.9861	0.9864	0.9706	0.9732	0.9778	0.9772	0.9083	0.9065	0.8944	0.9023	0.8941	0.8908
**3DHL_2B**	0.9800	0.9795	0.9988	1.0000	0.9808	0.9786	0.9863	0.9862	0.9711	0.9736	0.9778	0.9776	0.9105	0.9085	0.8965	0.9037	0.8957	0.8929
**3DHL_3A**	0.9892	0.9902	0.9813	0.9808	1.0000	0.9982	0.9720	0.9730	0.9692	0.9731	0.9773	0.9763	0.9471	0.9473	0.9392	0.9438	0.9409	0.9375
**3DHL_3B**	0.9928	0.9935	0.9788	0.9786	0.9982	1.0000	0.9716	0.9723	0.9704	0.9732	0.9773	0.9761	0.9497	0.9492	0.9401	0.9439	0.9460	0.9420
**3DH_1A**	0.9746	0.9760	0.9861	0.9863	0.9720	0.9716	1.0000	0.9986	0.9737	0.9753	0.9875	0.9872	0.8946	0.8936	0.8760	0.8828	0.8815	0.8754
**3DH_1B**	0.9755	0.9769	0.9864	0.9862	0.9730	0.9723	0.9986	1.0000	0.9750	0.9764	0.9887	0.9879	0.8966	0.8958	0.8786	0.8850	0.8823	0.8763
**3DH_2A**	0.9742	0.9748	0.9706	0.9711	0.9692	0.9704	0.9737	0.9750	1.0000	0.9983	0.9839	0.9848	0.9421	0.9416	0.9306	0.9343	0.9247	0.9181
**3DH_2B**	0.9755	0.9756	0.9732	0.9736	0.9731	0.9732	0.9753	0.9764	0.9983	1.0000	0.9848	0.9858	0.9432	0.9433	0.9319	0.9360	0.9262	0.9216
**3DH_3A**	0.9772	0.9784	0.9778	0.9778	0.9773	0.9773	0.9875	0.9887	0.9839	0.9848	1.0000	0.9982	0.9198	0.9198	0.9032	0.9084	0.9138	0.9088
**3DH_3B**	0.9774	0.9775	0.9772	0.9776	0.9763	0.9761	0.9872	0.9879	0.9848	0.9858	0.9982	1.0000	0.9222	0.9213	0.9049	0.9105	0.9157	0.9098
**HM_1A**	0.9496	0.9481	0.9083	0.9105	0.9471	0.9497	0.8946	0.8966	0.9421	0.9432	0.9198	0.9222	1.0000	0.9987	0.9912	0.9924	0.9826	0.9788
**HM_1B**	0.9466	0.9462	0.9065	0.9085	0.9473	0.9492	0.8936	0.8958	0.9416	0.9433	0.9198	0.9213	0.9987	1.0000	0.9925	0.9924	0.9807	0.9780
**HM_2A**	0.9354	0.9353	0.8944	0.8965	0.9392	0.9401	0.8760	0.8786	0.9306	0.9319	0.9032	0.9049	0.9912	0.9925	1.0000	0.9982	0.9759	0.9721
**HM_2B**	0.9398	0.9391	0.9023	0.9037	0.9438	0.9439	0.8828	0.8850	0.9343	0.9360	0.9084	0.9105	0.9924	0.9924	0.9982	1.0000	0.9778	0.9733
**HM_3A**	0.9471	0.9450	0.8941	0.8957	0.9409	0.9460	0.8815	0.8823	0.9247	0.9262	0.9138	0.9157	0.9826	0.9807	0.9759	0.9778	1.0000	0.9953
**HM_3B**	0.9424	0.9406	0.8908	0.8929	0.9375	0.9420	0.8754	0.8763	0.9181	0.9216	0.9088	0.9098	0.9788	0.9780	0.9721	0.9733	0.9953	1.0000

KEY: **3DHL_1A**

Technical replicate (A/B)

Construct

Biological replicate (1/2/3)

Color scale 0.876 → 1.000

Averaged Pearson Correlations Between Constructs

HM/3DHL	0.9291
3DHL/3DH	0.9761
HM/3DH	0.9103

Averaged Pearson Correlations Between Replicates Within Constructs

Construct	Biological	Technical
3DHL	0.9835	0.9985
3DH	0.9826	0.9984
HM	0.9823	0.9974

Table S1. Pearson Coefficients Across Model Configurations and Replicates. The first column and row indicate individual LC-MS runs organized by replicates. Biological replicates are separate constructs (in triplicate) and technical replicates are duplicate injections of the same sample (see Key). Averaged Pearson Correlations between constructs and replicates are shown to the right of the Key.

Table S2: DAVID Analysis of Enriched KEGG pathways. Terms exhibiting p-values less than 0.05 are shown. Arranged alphabetically according to the KEGG pathway. Multiple entries are for separate pairwise comparisons. p-values < 0.0001 (1.0E-04) are shown in bold. Pathways with over 200 proteins were excluded from the list.

KEGG pathway	KEGG ID	Enrichment	Comparison	Proteins Annotated	Proteins in pathway	p-value
alpha-Linolenic acid metabolism	Map00592	3DH	3DH vs HM	3	25	3.35E-02
Arginine and proline metabolism	Map00330	3DH	3DH vs HM	5	51	1.66E-03
Arginine and proline metabolism	Map00330	3DHL	3DHL vs HM	4	51	2.21E-02
Ascorbate and aldarate metabolism	Map00053	3DHL	3DHL vs HM	6	27	**1.53E-05**
Ascorbate and aldarate metabolism	Map00053	3DH	3DH vs HM	5	27	1.52E-04
beta-Alanine metabolism	Map00410	3DH	3DH vs HM	6	34	**1.56E-05**
beta-Alanine metabolism	Map00410	3DHL	3DHL vs HM	6	34	**4.62E-05**
Biosynthesis of amino acids	Map01230	HM	3DHL vs HM	11	83	**3.28E-08**
Biosynthesis of amino acids	Map01230	HM	3DH vs HM	11	83	**3.76E-07**
Biosynthesis of amino acids	Map01230	3DH	3DH vs HM	5	83	7.45E-03
Biosynthesis of unsaturated fatty acids	Map01040	3DH	3DH vs HM	4	29	3.59E-03
Biosynthesis of unsaturated fatty acids	Map01040	3DHL	3DHL vs HM	4	29	5.45E-03
Butanoate metabolism	Map00650	3DHL	3DHL vs HM	7	28	**6.05E-07**
Butanoate metabolism	Map00650	3DH	3DH vs HM	6	28	**7.56E-06**
Carbon metabolism	Map01200	3DHL	3DHL vs HM	13	121	**2.00E-09**
Carbon metabolism	Map01200	HM	3DHL vs HM	13	121	**8.04E-09**
Carbon metabolism	Map01200	HM	3DH vs HM	14	121	**1.69E-08**
Carbon metabolism	Map01200	3DH	3DH vs HM	9	121	**7.44E-06**
Chemical carcinogenesis	Map05204	3DHL	3DHL vs HM	6	91	3.21E-03
Chemical carcinogenesis	Map05204	HM	3DH vs HM	7	91	5.21E-03
Citrate cycle (TCA cycle)	Map00020	3DHL	3DHL vs HM	5	32	5.77E-04
Citrate cycle (TCA cycle)	Map00020	HM	3DH vs HM	4	32	4.42E-02
Drug metabolism - cytochrome P450	Map00982	3DHL	3DHL vs HM	5	71	8.13E-03
Drug metabolism - cytochrome P450	Map00982	HM	3DH vs HM	6	71	9.20E-03
Drug metabolism - cytochrome P450	Map00982	3DH	3DH vs HM	4	71	3.42E-02
Drug metabolism - other enzymes	Map00983	3DHL	3DHL vs HM	6	56	4.40E-04
Fatty acid degradation	Map00071	3DHL	3DHL vs HM	16	47	**1.67E-19**
Fatty acid degradation	Map00071	3DH	3DH vs HM	13	47	**4.00E-15**
Fatty acid elongation	Map00062	3DHL	3DHL vs HM	5	29	4.44E-04
Fatty acid metabolism	Map01212	3DHL	3DHL vs HM	11	54	**1.67E-10**
Fatty acid metabolism	Map01212	3DH	3DH vs HM	9	54	**2.21E-08**
Fructose and mannose metabolism	Map00051	HM	3DH vs HM	5	37	7.46E-03
Glutathione metabolism	Map00480	HM	3DH vs HM	8	58	**4.50E-05**
Glycine, serine and threonine metabolism	Map00260	3DHL	3DHL vs HM	6	39	**8.55E-05**
Glycine, serine and threonine metabolism	Map00260	3DH	3DH vs HM	4	39	7.69E-03
Glycolysis / Gluconeogenesis	Map00010	HM	3DHL vs HM	11	70	**9.71E-09**
Glycolysis / Gluconeogenesis	Map00010	HM	3DH vs HM	8	70	1.39E-04
Glyoxylate and dicarboxylate metabolism	Map00630	3DH	3DH vs HM	4	27	3.26E-03
Glyoxylate and dicarboxylate metabolism	Map00630	3DHL	3DHL vs HM	4	27	4.63E-03
Histidine metabolism	Map00340	3DH	3DH vs HM	3	24	3.36E-02
Lysine biosynthesis	Map00300	3DH	3DH vs HM	2	2	3.60E-02
Lysine biosynthesis	Map00300	3DHL	3DHL vs HM	2	2	4.05E-02
Lysine degradation	Map00310	3DHL	3DHL vs HM	10	52	**2.38E-09**
Lysine degradation	Map00310	3DH	3DH vs HM	7	52	**7.58E-06**
Metabolism of xenobiotics by cytochrome P450	Map00980	3DHL	3DHL vs HM	5	70	8.05E-03
Metabolism of xenobiotics by cytochrome P450	Map00980	HM	3DH vs HM	6	70	9.41E-03
Pentose and glucuronate interconversions	Map00040	3DHL	3DHL vs HM	5	36	8.68E-04
Pentose and glucuronate interconversions	Map00040	3DH	3DH vs HM	4	36	6.42E-03
Pentose phosphate pathway	Map00030	HM	3DHL vs HM	7	29	**2.31E-06**
Pentose phosphate pathway	Map00030	HM	3DH vs HM	6	29	1.78E-04
Peroxisome	Map04146	3DHL	3DHL vs HM	14	85	**1.75E-12**
Peroxisome	Map04146	3DH	3DH vs HM	13	85	**2.67E-12**
Porphyrin and chlorophyll metabolism	Map00860	3DHL	3DHL vs HM	4	41	1.30E-02
PPAR signaling pathway	Map03320	3DH	3DH vs HM	8	77	**5.86E-06**
PPAR signaling pathway	Map03320	3DHL	3DHL vs HM	6	77	1.57E-03
Primary bile acid biosynthesis	Map00120	3DH	3DH vs HM	4	16	7.52E-04
Primary bile acid biosynthesis	Map00120	3DHL	3DHL vs HM	4	16	1.11E-03

Propanoate metabolism	Map00640	3DHL	3DHL vs HM	7	28	**6.05E-07**
Propanoate metabolism	Map00640	3DH	3DH vs HM	4	28	3.42E-03
Protein processing in endoplasmic reticulum	Map04141	3DHL	3DHL vs 3DH	5	168	5.34E-04
Pyruvate metabolism	Map00620	3DH	3DH vs HM	4	40	7.52E-03
Retinol metabolism	Map00830	3DHL	3DHL vs HM	5	83	1.32E-02
Retinol metabolism	Map00830	3DHL	3DHL vs 3DH	3	83	2.79E-02
Ribosome	Map03010	HM	3DHL vs HM	18	172	**3.68E-12**
Ribosome	Map03010	HM	3DH vs HM	15	172	**8.88E-08**
Thyroid hormone synthesis	Map04918	3DHL	3DHL vs 3DH	3	68	2.83E-02
Tryptophan metabolism	Map00380	3DHL	3DHL vs HM	10	46	**9.52E-10**
Tryptophan metabolism	Map00380	3DH	3DH vs HM	7	46	**5.13E-06**
Valine, leucine and isoleucine degradation	Map00280	3DHL	3DHL vs HM	17	55	**6.12E-20**
Valine, leucine and isoleucine degradation	Map00280	3DH	3DH vs HM	13	55	**1.58E-14**

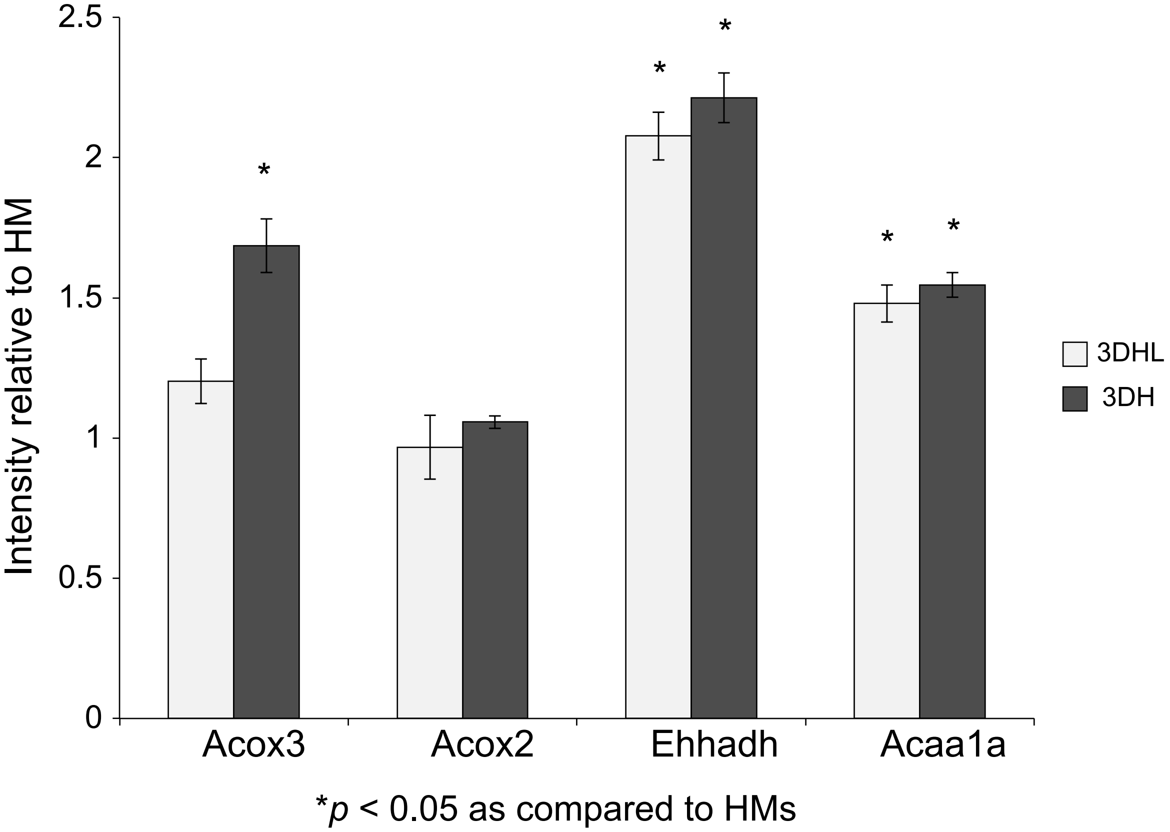

Fig S2. Peroxisomal processes are modulated by PEMs and LSECs. Relative protein abundance ratios based on the Top3 ion intensities for enzymes associated with peroxisomal fatty acid β-oxidation that change significantly based upon the culture conditions (* $p < 0.05$ as compared to HMs).
